# Spatiotemporal analysis of the effect of global development indicators on child mortality

**DOI:** 10.1186/s12942-023-00330-x

**Published:** 2023-05-04

**Authors:** Prince M. Amegbor, Angelina Addae

**Affiliations:** 1 Global and Environmental Public Health, School of Global Public Health, New York University, 708 Broadway, New York, NY 10003 USA; 2 Department of Economics, University of Saskatchewan, 129, 72 Campus Drive, Saskatoon, SK S7N 5B5 Canada

## Abstract

**Background:**

Child mortality continue to be a major public health issue in most developing countries; albeit there has been a decline in global under-five deaths. The differences in child mortality can best be explained by socioeconomic and environmental inequalities among countries. In this study, we explore the effect of country-level development indicators on under-five mortality rates. Specifically, we examine potential spatio-temporal heterogeneity in the association between major world development indicators on under-five mortality, as well as, visualize the global differential time trend of under-five mortality rates.

**Methods:**

The data from 195 countries were curated from the World Bank’s World Development Indicators (WDI) spanning from 2000 to 2017 and national estimates for under-five mortality from the UN Inter-agency Group for Child Mortality Estimation (UN IGME).We built parametric and non-parametric Bayesian space-time interaction models to examine the effect of development indicators on under-five mortality rates. We also used employed Bayesian spatio-temporal varying coefficient models to assess the spatial and temporal variations in the effect of development indicators on under-five mortality rates.

**Results:**

In both parametric and non-parametric models, the results show indicators of good socioeconomic development were associated with a reduction in under-five mortality rates while poor indicators were associated with an increase in under-five mortality rates. For instance, the parametric model shows that gross domestic product (GDP) (β = − 1.26, [CI − 1.51; − 1.01]), current healthcare expenditure (β = − 0.40, [CI − 0.55; − 0.26]) and access to basic sanitation (β = − 0.03, [CI − 0.05; − 0.01]) were associated with a reduction under-five mortality. An increase in the proportion practising open defecation (β = 0.14, [CI 0.08; 0.20]) an increase under-five mortality rate. The result of the spatial components spatial variation in the effect of the development indicators on under-five mortality rates. The spatial patterns of the effect also change over time for some indicators, such as PM2.5.

**Conclusion:**

The findings show that the burden of under-five mortality rates was considerably higher among sub-Saharan African countries and some southern Asian countries. The findings also reveal the trend in reduction in the sub-Saharan African region has been slower than the global trend.

**Supplementary Information:**

The online version contains supplementary material available at 10.1186/s12942-023-00330-x.

## Background

The global burden of child mortality is disproportionate high among countries with limited resources or the so-called “low- and middle-income countries” (LMIC). For instance, in 2018 the under-five mortality rate (U5MR) in the Sub-Saharan African region was approximately twice (78 per 1000 live births) the global rate of 39 deaths per 1000 live births [1]. The United Nations’ Sustainable Development Goals number 3 and 10 advocate for improving and promoting healthy lives and wellbeing, and reducing disparities in global health indicators among (and within) countries, respectively [2]. Although the disproportionate disparities in global U5MR paints a grim picture, estimates from studies show a significant decline in all regions [1, 3]. Global U5MR declined from 93.0 deaths per 1000 live births in 1990 to 37.7 in 2019, representing 59% decline in global U5MR [3]. In sub-Saharan Africa and South Asia, U5MR reduced from 178.5 and 129.5, respectively, in 1990 to 75.8 and 40.2, respectively, in 2019 [3]. Disparities in regional and global U5MR are mainly attributed to differences in socioeconomic development and environmental exposures [4–8]

Socioeconomic gradient in health and wellbeing is a ubiquitous in public health. The evidence in existing studies show that persons of low socioeconomic status have higher morbidity and mortality compared to those of higher socioeconomic position [4, 6, 9]. Likewise, persons of poor socioeconomic background have higher burden of environmental risk exposures [10, 11]. In resource-limited countries, including those in sub-Saharan Africa and southern Asia, the majority of U5MR is preventable and caused by penury-related factors [1, 12]. For instance, 45% of U5MR is attributable to poor dietary lifestyle and the inadequate access to nutritious food—undernutrition [13]. Children in poor socioeconomic settings often have limited access to food rich in essential micronutrients, thus, increasing the vulnerability to diseases and increased risk of death. Approximately 5.3% of U5MR deaths are due to inadequate access to clean drinking water, poor sanitation and hygiene [14, 15]. A quarter of U5MR deaths is due to environmental risk exposures and in 2016 air pollution accounted for 543,000 deaths among children under age 5 years [16].

Increasingly, the research shows that investments aimed at improving socioeconomic status and reducing environmental risk exposures reduce U5MR. For instances, findings from studies in Malawi, Uganda, India, Nigeria, and other developing countries have found evidence for a positive causal relationship between education and child mortality [17, 18]. The research shows that 12 years of maternal and paternal education is associated with 31% and 17%, respectively, reduction in U5MR [9]. Higher parental education often translates into quality health and healthcare seeking behaviour, health literacy and healthy feeding practices that improves the health and wellbeing of children. Increasing public funding for health and healthcare is also known to reduce the incidence of child and infant mortality, especially in sub-Saharan Africa [19, 20]. When investments in healthcare and services are done in fair and equitable manner, they improve access to vital care services, including pediatric care, for the needy and vulnerable populations. Likewise, the evidence from existing studies point to strong correlation between economic growth indicators and child mortality. Countries with higher Gross Domestic Product (GDP) and lower unemployment rates generally have lower child mortality [6, 21]. An empirical analysis study conducted in South Asia revealed that per-capita income and urbanization play a vital role in the reduction of child mortality [22]. Other studies conducted in Greece, South Africa, sub-Saharan Africa, Brazil, and across the world show similar conclusions that economic growth reduces child mortality [23–26]. Access and use of improved sanitation coupled with good hygiene also reduces the risk or susceptibility to diseases hence improve the quality of life of children. There is a plethora of evidence that shows that good sanitation and hygiene have a negative correlation with child mortality [27–29].

Nonetheless, there is limited research on the spatio-temporal effect of socioeconomic development and environmental indicators on global U5MR. Existing studies have also failed to explore potential spatial and temporal heterogeneity in the association between these development and environmental indicators and U5MR. In order to meet the Sustainable Development Goals (SDGs), particularly improve health and reduce spatial inequalities in health, there is the need to incorporate spatial-temporal perspectives in the current discourse on global health. In view of the current gap in the research, our study seeks to explore the spatio-temporal effect of global development indicators on U5MR. Specifically, the study sought to:

1. Examine the spatio-temporal effects of key global development indicators on under-five rates among countries of the world.
2. Determine if there are spatial and temporal variations in the association between the development indicators and under-five rates.
3. Map and visualize these spatial and temporal differences in the association between key global development indicators and U5MR.

## Data and methods

The data from this study was derived from the World Bank’s World Development Indicators (WDI). The WDI data contains statistical information on major global development indicators compiled from officially-recognised international sources, including national statistics bureaus. The WDI data is available at national, regional and global levels. Given the focus of our study, we used the national estimates from 195 countries. The original data had information on 217 countries and territories. In this study we excluded territories of sovereign states; that is, data on territories such as the Faroe Islands and the British Virgin Islands were excluded from the study. A full list of the countries used in this study can be found in the supplementary material. The WDI data used in this study span from 2000 to 2017.

The outcome variable of interest in this study was under-five mortality rate per 1,000 live births. The national estimates for under-five mortality rates were compiled by the UN Inter-agency Group for Child Mortality Estimation (UN IGME) which consists of the following international organisation: the United Nations Children’s Fund (UNICEF), the World Health Organization (WHO), the World Bank and the Population Division of the United Nations’ Department of Economic and Social Affairs (UN DESA Population). The UN IGME under-five mortality rate estimates are based on nationally representative data, including data sourced from household surveys and censuses. To ensure quality data (estimates), the UN IGME excludes data sources with substantial non-sampling errors or omissions from the statistical model used to derive the estimates. The UN IGME estimation process does not use covariates and a detailed description of the modelling process can be found elsewhere [30].

Evidence from existing studies highlight correlation between national socioeconomic development and child health [31–33]. The research shows that contextual socioeconomic, environmental and political factors have significant impact on the health and wellbeing of children [33–35]. In view of this, we used socioeconomic and environmental indicators from the World Bank’s WDI as predictors of under-five mortality rate per 1,000 live births. The WDI has 1,442 indictors or measures grouped under 12 major domains (Economic Policy and Debt, Education, Environment, Health, Gender, Financial Sector, Infrastructure, Poverty, Private Sector and Trade, Social, Public Sectors, Social Protection and Labor). Based on the evidence from existing studies, we used nine major global development indicators as determinants or predictors of under-five mortality rate per 1,000 live births. The covariates used in this study were:

- Percentage of females with secondary education
- Percentage of females aged 15 years and above employed as a ratio of the total population
- Gross domestic product—GDP—per capita (current US$)
- Gross national expenditure as a percentage of GDP
- Percentage of out-of-pocket expenditure out of current health expenditure
- Percentage of people using at least basic drinking water services
- Percentage of the population practising open defecation
- Percentage of population using at least basic sanitation services
- Mean annual PM2.5 exposure in micrograms per cubic meter

## Analysis

### Spatio-temporal analysis

First, we examined the association between the world development indicators used in this study and under-five mortality rate per 1000 live births. Considering the temporal dimension of the data, we employed Bayesian Spatio-temporal modelling to assess the effect of the development indicators on under-five mortality. Several space-time interaction Bayesian models for disease risk and mapping have been proposed in existing literature [36]. These models can be broadly classified into the parametric and non-parametric trends for the temporal component. These models conceptualise the nature of interaction between space and time. We employed these models to assess the best space-time interaction models in relations to the study objectives. The model for the parametric trend for the temporal component (Model 1) was proposed by Bernardinelli et al. [37]. The proposed model encapsulates the main spatial effects for the entire time period and a linear time trend. Considering our outcome (under-five mortality rate), Bernardinelli et al. [37] proposed model can be expressed as:

1 $$\eta_{it} = b_{0} + u_{i} + v_{i} + \left( {\beta + \delta_{i} } \right) \times t$$

$$y_{it} \sim {\text{Normal}}\left( {\eta_{it} ,\sigma_{e}^{2} } \right)$$

where ${y}_{it}$ is the under-five mortality rate for country i and year t; ${\sigma }_{e}^{2}$ is the variance of the measurement error defined by a Gaussian white-noise process (serially and spatially uncorrelated); ${b}_{0}$ is the intercept; ${u}_{i}and {v}_{i}$ are the Besag-York-Mollie (BYM) specification for the spatially structured residual (effect) and unstructured residual (effect), respectively. $\beta$ represents the global time effect and a differential time trend which identifies the interaction between time and space is given as ${\delta }_{i}$. ${\eta }_{it}$ is the linear predictor for response Y. For the differential time trend, when the ${\delta }_{i}$ is less than 0 it signifies the trend is less steep compared to the average trend while ${\delta }_{i}$ values greater than 0 signifies a steeper trend compared to the average [36, 38]

For the non-parametric models, five models with different space-time interaction specifications were built. The first non-parametric model (Model 2) is similar to Bernardinelli et al. [37] proposed model (Model 1), however, it drops the linearity constraint imposed on the differential temporal trend through a non-parametric formulation for the linear predictor as proposed by Knorr-Held [39]. This can be expressed as:

2 $$\eta_{it} = b_{0} + u_{i} + v_{i} + \gamma_{t} + \phi_{t}$$

where ${b}_{0},{u}_{i, }and {v}_{i}$ are the same as Eq. (1) and ${\gamma }_{t}$ is the temporally structured effect, model using a random walk order of 2 (RW2). RW2 is defined as”

3 $$\gamma_{t} \left| {\gamma_{t - 1} } \right.,\,\gamma_{t - 2} \, \sim {\text{Normal}}\,\left( {2\gamma_{t - 1} + \,\,\gamma_{t - 2} ,\sigma^{2} } \right)$$

${\phi }_{t}$ is an unstructured temporal effect specified by means of a Gaussian exchangeable prior, given as:

4 $$\phi_{t} \sim {\text{Normal}}\left( {0,1/\tau_{\phi } } \right)$$

The remaining four models are extensions of Model 2 that allow four different types of interactions between space and time as proposed by Knorr-Held (2000). The first space-time interaction (TYPE I) is based on the assumption of unstructured space-time effect; that is, neighbouring spatial units do not affect each other and mortality rates of previous years do not affect that of subsequent years—Model 3. The second space-time interaction (TYPE II) assumes an interaction between a structured temporal effect and an unstructured spatial effect—Model 4. The third space-time interaction (TYPE III) is based on the assumption of an interaction between an unstructured temporal effect and a spatially structured effect—Model 5. Finally, the fourth space-time interaction (TYPE IV) is a complex interaction model that assumes an interaction between spatially and temporally structured effects—Model 6. A detailed discussion of these four space-time interactions and their implementations are described elsewhere [36, 38, 39].

### Spatiotemporally varying coefficients (STVC) analysis

Next, a Bayesian spatiotemporally varying coefficient (or Bayesian spatial heterogeneity) model to explore spatial and temporal variations in the relationships between the development indicators and under-five mortality rates. Compared to traditional Spatio-temporal models, STVC models move beyond the naïve assumption that the association between predictors and outcome are the same across space and time. It also accounts for autocorrelation in the spatial and temporal dimension of the data; that is, neighbouring units have the potential of affecting the outcome in a unit and mortality rates of previous time periods affect the rate of successive periods. The Bayesian STVC model can be defined simply as:

$$\eta_{i,t} = g\left( {Y_{i,t} } \right) = \alpha + f\left( {{\varvec{\beta}}_{i,t}^{^{\prime}} {\mathbf{x}}_{i,t} } \right)$$

where ${\eta }_{i,t}$ is the linear predictor for response Y (under-five mortality rate) for country i at time t, given *i* = 1, 2 …, n and *t* = 1,2,3, …, T; and g(.) is the appropriate link function. $\alpha$ is the intercept, ${{\varvec{\beta}}}_{i,t}^{\mathrm{^{\prime}}}$ is the local coefficient for country i at time t (or the local-scale spatial and temporal coefficients) spatial and temporal, and ${\mathbf{x}}_{i,t}$ is the covariate matrix for the development indicators in the i-th country at the t-th year. The intrinsic Gaussian Markov random field (IGMRF) for the spatiotemporally varying random effects for estimating the local (country-level) coefficient at each time is given as $f$(). In this model, we assumed a “besag” model; that is, the spatial random effect is assigned the intrinsic conditional autoregressive (iCAR) distribution for the smoothing of the data according to a defined neighbourhood structure. The iCAR distribution is based on the geographic concept of spatial dependence, that is, the association between the covariates and outcome in a given location (or country) are similar to that of neighbouring locations (or countries)—geographically autocorrelated. An autoregressive model of order 1 was adopted for the temporal structured random effects. A detailed description of the Bayesian STVC model formulation is described elsewhere [40].

Both Spatio-temporal and Spatiotemporally varying coefficients models were implemented using the integrated nested Laplace approximations (INLA) framework with the PARDISO sparse matrix library for high-performance computing [41]. INLA is a computationally less-intensive deterministic algorithm for Bayesian inference based on the latent Gaussian model (LGMs). The default prior specification of R-INLA was used for the distribution of the hyperparameters—a log gamma prior with parameters a = 0.001 and b = 0.001 We assumed a vague prior with parameters a = 1 and b = 0.00005. In the Bayesian models, the parameter estimates were considered significant if the lower and upper 95% credible intervals (Crl) show the same direction for the association. That is, both lower and upper credible intervals are above zero or below zero. All analyses were modelled in the open-access R software [42] using the R-INLA package [43–45]. Results of the Spatio-temporal and spatiotemporally varying coefficient models were also visualised in R software using the tmap and tmaptools packages [46]. Information on missing data and the distribution of missingness can be found in the supplementary material (Additional file 1: Tables S2 and S4).

### Model selection

For the Spatio-temporal models, we compared the results of the Bernardinelli et al. [37] parametric trend for the temporal component and the results for the non-parametric trend models. The optimal model choice for the non-parametric temporal trend models was determined by the deviance information criterion (DIC) and the Watanabe-Akaike information criterion (WAIC) values. DIC and WAIC are criteria for model assessment and model choice. DIC and WAIC as model assessment criteria accounts for both the goodness-of-fit and the complexity of the model through the estimated effective number of parameters [47]. The difference between the two lies in how the effective number of parameters is computed. A detailed description of the DIC and WAIC as measures of model fit can be found elsewhere [38, 47, 48]. Table 1 below provides the model fit information for the Spatio-temporal models. Similarly, to the Akaike information criterion (AIC), smaller DIC and WAIC values indicate a better model fit for the data. The DIC (17889.79) and WAIC (18108.96) show that for the non-parametric temporal trend models, the model with Type IV (Model 6) space-time interaction is a better fit or statistically preferable compared to the others. The DIC and WAIC values also show that the parametric temporal trend model (Model 1) is a better fit than all non-parametric temporal trend models, except Model 6 (model with Type IV space-time interaction).

**Table 1:** Diagnostics information for space-time interaction model selection

	DIC	WAIC
Model 1—Parametric model (Bernardinelli model)	18657.42	18897.03
Model 2—Non-parametric temporal effect	25563.05	25588.74
Model 3—TYPE I	25211.67	25353.74
Model 4—TYPE II	38085.58	38085.57
Model 5—TYPE III	25604.86	25680.23
Model 6—TYPE IV	17889.79	18108.96

## Results

### Descriptive

Table 2 below provides the summary statistics of the study variables. The mean global under-five mortality rate per 1000 live births for the 18 years temporal coverage was 41.3. Also, within the study period, the percentage of female secondary education pupils was 48.1%. The mean percentages of people with access to basic drinking water and basic sanitation services were 84.3% and 71.2%, respectively. The result indicates an average of 10.8% of the global population practised open defecation in the study period (2000–2017); while the mean annual PM2.5 exposure was 9.3 µgm^−3^. Additional file 1: Table S1 in the supplementary material provides a summary statistics of study variables by countries.

**Table 2:** Descriptive summary of study variables

Variables	Mean	Range	1st Quantile–3rd Quantile
Mortality rate, under-5 (per 1,000 live births)	41.3	1.8–234.0	9.1–62.6
Secondary education, pupils (% female)	48.1	0.0–58.1	47.7–50.3
Employment to population ratio, 15 + , female (%) (ILO estimate)	47.1	4.5–86.0	37.9–57.6
Gross domestic product—per capita (current US$)	12,794.1	111.9–189,422.2	1,244.4–14,092.2
Gross national expenditure (% of GDP)	105.6	45.3–264.8	96.7–113.2
Out-of-pocket expenditure (% of current health expenditure)	34.4	0.1–87.1	17.9–48.5
Current health expenditure (% of GDP)	6.3	1.0–25.5	4.3–8.0
People using at least basic drinking water services (% of population)	84.3	18.7–100.0	75.4–99.0
People practicing open defecation (% of population)	10.8	0.0–84.6	0.0–13.7
People using at least basic sanitation services (% of population)	71.2	3.4–100.0	45.9–97.7
PM2.5 air pollution, mean annual exposure (micrograms per cubic meter)	9.3	− 0.3–71.5	3.2–13.1

### Spatio-temporal model

This section presents the results of the parametric temporal trend and the Type IV space-time interaction models. The estimates of the fixed-effect component of the model for the parametric temporal trend model (hereinafter called parametric model) and Type IV space-time interaction model (Model 6) are shown in Table 3 below. For the parametric model (Model 1), the result shows that GDP (β = -1.26, [CrI = − 1.51; − 1.01]), current health expenditure (β = − 0.40, [CrI = − 0.55; − 0.26]) and access to basic sanitation services (β = − 0.03, [CrI = − 0.05; − 0.01]) were associated with a reduction in the under-five mortality rate. Likewise, for the parametric time, the model shows that under-five mortality rates reduce over time (β = − 1.48, [CrI = − 1.74; − 1.21]). An increase in the percentage of the population practising open defecation was associated with an increase in under-five mortality rates (β = 0.14, [CrI = 0.08; 0.20]). In Model 6 (Type IV assuming interaction between spatially and temporally structured effects), the result of the parameter estimates shows that an increase in the proportion of female secondary education pupils (β = − 0.02, [CrI = − 0.02; − 0.01]), GDP (β = − 0.47, [CrI = − 0.82; − 0.12]), access to basic drinking water (β = − 0.14, [CrI = − 0.17; − 0.11]), increased access to sanitation (β = − 0.04, [CrI = − 0.07; − 0.01]) are associated with a reduction in under-five mortality rates. On the other hand, the percentage of employed females (β = 0.21, [CrI = 0.10; 0.33]) and people practising open defecation (β = 0.13, [CrI = 0.06; 0.20]) were associated with an increase in under-five mortality rates.

**Table 3:** Results of the effect estimates of development indicators on under five mortality rates from the parametric and non-parametric space-time models

	Model 1		Model 6 (Type IV Interaction)	
	Mean	95% Cred. Interval	Mean	95% Cred. Interval
Fixed Effect Component				
Intercept	66.28	58.26 to 74.29	38.72	32.95 to 44.49
Secondary education, pupils (% female)	− 0.01	− 0.02 to 0.00	− **0.02**	− **0.02 to **− **0.01**
Employment to population ratio, 15 + , female (%) (ILO estimate)	0.09	− 0.01 to 0.18	**0.39**	**0.34 to** **0.45**
Gross domestic product—per capita (natural logarithm scale)	− **1.26**	**− 1.51 to − 1.01**	− **0.47**	− **0.82 to **− **0.12**
Gross national expenditure (% of GDP)	0.01	− 0.00 to 0.02	− 0.00	− 0.01 to 0.01
Out-of-pocket expenditure (% of current health expenditure)	− 0.01	− 0.03 to 0.02	− 0.01	− 0.05 to 0.02
Current health expenditure (% of GDP)	− **0.40**	**− 0.55 to − 0.26**	**0.27**	**0.10 to ** **0.44**
People using at least basic drinking water services (% of population)	− 0.02	− 0.05 to 0.00	− **0.14**	− **0.17 to **− **0.11**
People practicing open defecation (% of population)	**0.14**	**0.08 to** **0.20**	**0.19**	**0.12 to** **0.26**
People using at least basic sanitation services (% of population)	− **0.03**	− **0.05 to **− **0.01**	− **0.04**	− **0.07 to** **-0.01**
PM2.5 air pollution, mean annual exposure (micrograms per cubic meter)	0.01	− 0.05 to 0.07	− **0.09**	− **0.11 to **− **0.02**
Year	− **1.48**	− **1.74 to** − **1.21**	–	–

Significant (95% Credible Interval) parameter estimates boldened

**Fig. 1:**
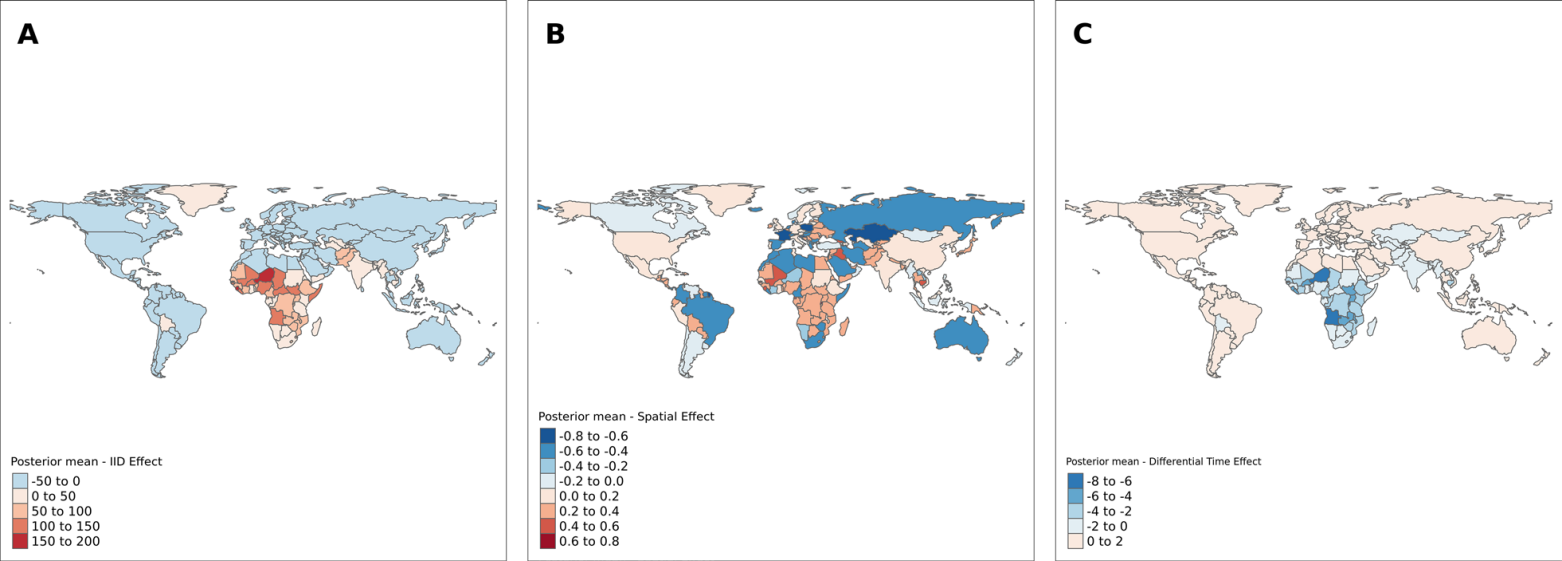
Country-specific unstructured spatially effect (IID), spatially structured (ICAR), and estimated differential time trend for under-five mortality rates—for Parametric model (Model 1)

Figure 1 below shows the results for the spatially structured, unstructured and differential time trend for under-five mortality by countries in the world. Panel A shows the spatial unstructured effect or country effect on under-five mortality. The map indicates that the country-specific effect of under-five mortality is higher among sub-Saharan African countries and some southern Asian countries. The effect of neighbouring countries on under-five mortality (spatially structured effect) is shown in Panel B. The results show heterogeneity in the distribution of high and low spatial effects across the globe; albeit, most countries in Africa and Asia show high spatial effects or effects from neighbouring countries on under-five mortality. The result for the differential time trend (${\delta }_{i}$)—interaction between time and space—is shown in Panel C. The result indicates that the differential time trend in under-five mortality rates in sub-Saharan African countries and some southern Asian countries is less steep compared to the average trend.

**Fig. 2:**
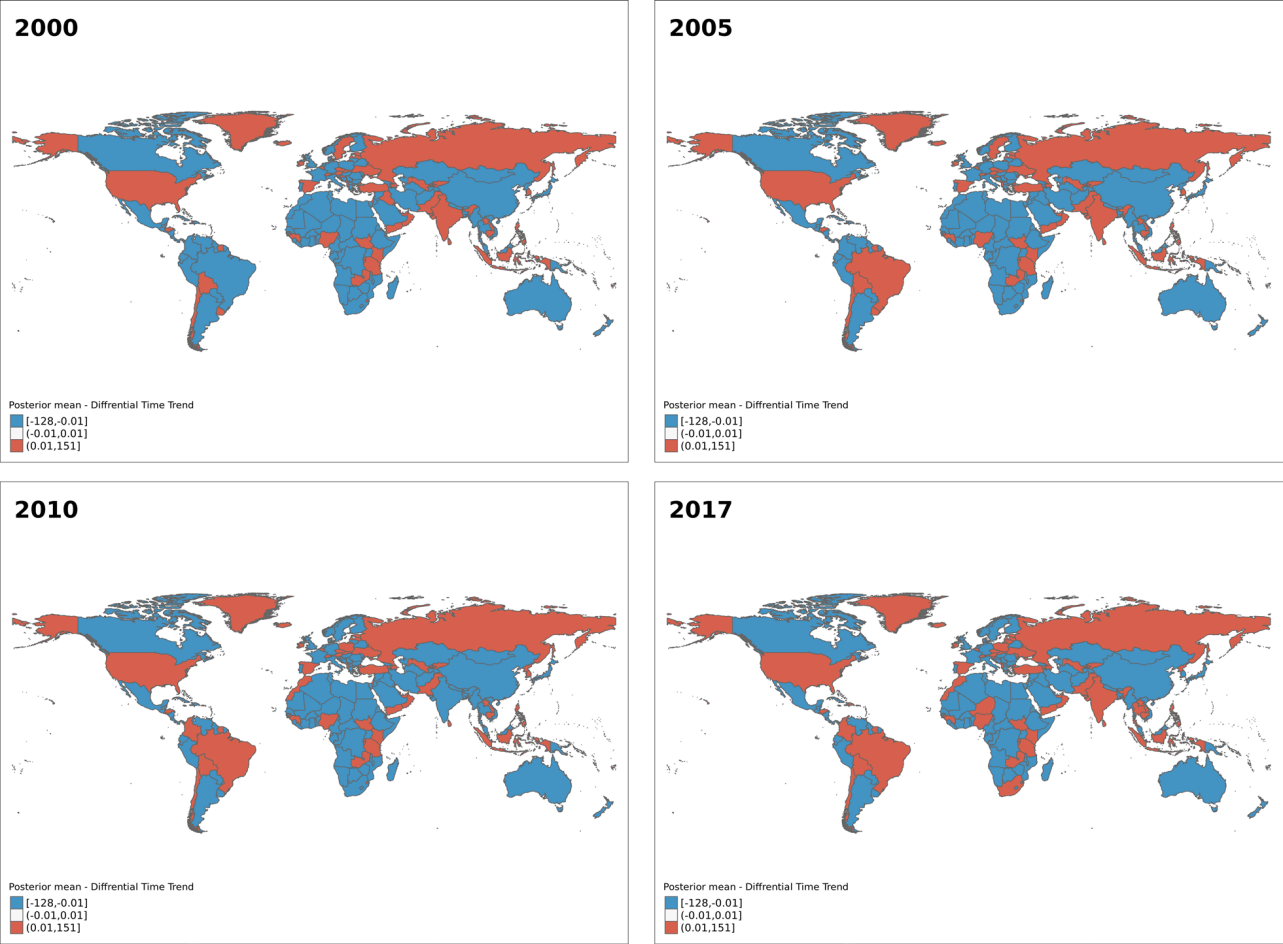
Estimated differential time trend for under-five mortality rates for the non-parametric model with TYPE IV space-time interaction (Model 6)

The differential trend for the space-time interaction for Model 6 for selected years is shown in Fig. 2 below. Similar to the differential trend for the parametric model, values less than zero signify a less steep trend compared to the average while values greater than zero indicate otherwise. The figures indicate the differential trend in most countries in Africa over the year is generally less steep compared to the average trend. We also observed a steeper trend in southern Africa in later years (from 2012 to 2017). Similar, trends are also evident in some countries in other parts of the world, including Europe, Asia and the Americas.

### Spatio-temporal varying coefficient model

**Fig. 3:**
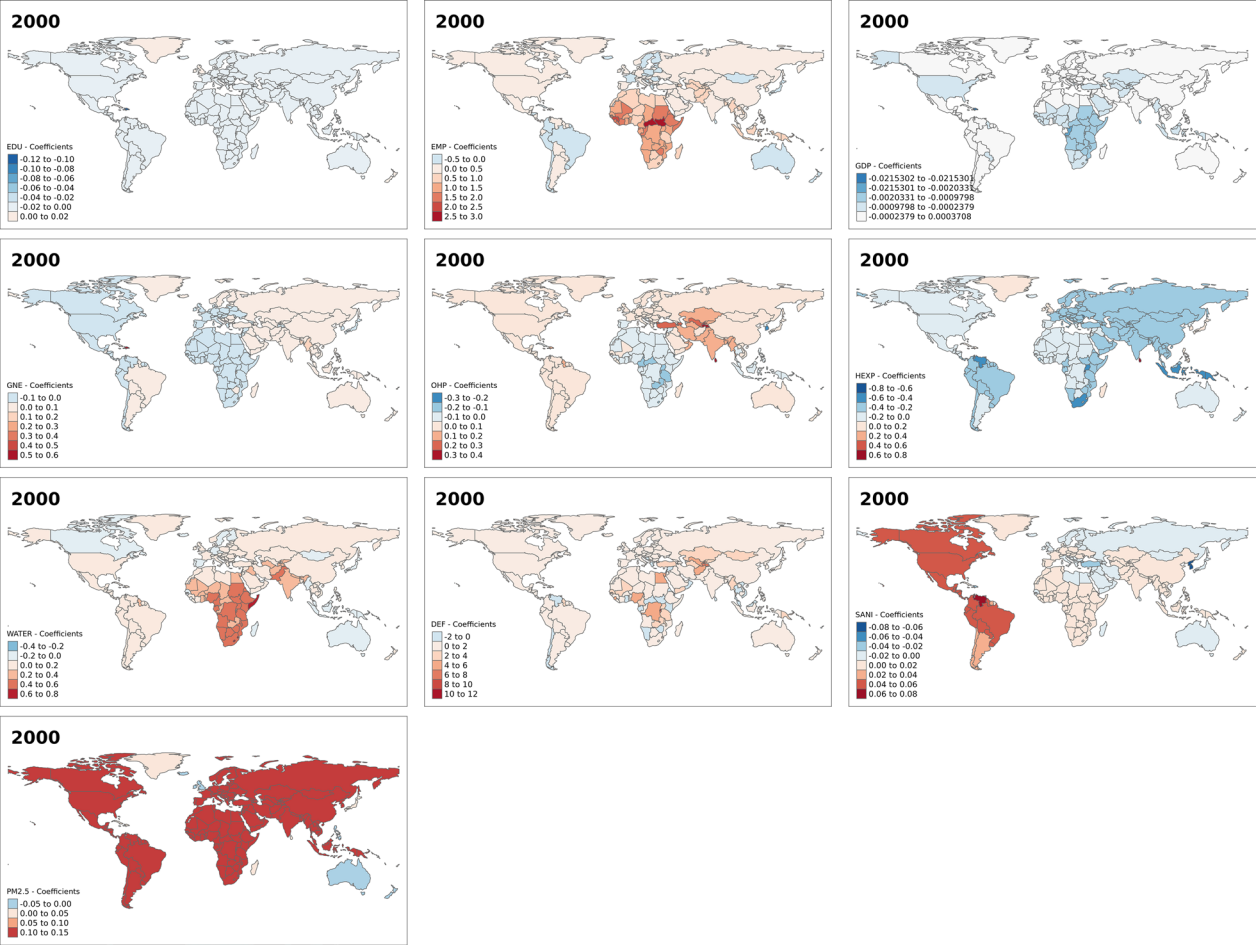
Spatial varying coefficients for the effects of development indicators on under five mortality rates in year 2000. EDU (Secondary education, pupils (% female)); EMP (Employment to population ratio, 15 + , female (%) (ILO estimate)); GDP (Gross domestic product—per capita (current US$)); GNE (Gross national expenditure (% of GDP)); OHP (Out-of-pocket expenditure (% of current health expenditure)); HEXP (Current health expenditure (% of GDP)); WATER (People using at least basic drinking water services (% of population)); DEF (People practicing open defecation (% of population)); SANI (People using at least basic sanitation services (% of population)); PM (PM2.5 air pollution, mean annual exposure (micrograms per cubic meter))

**Fig. 4:**
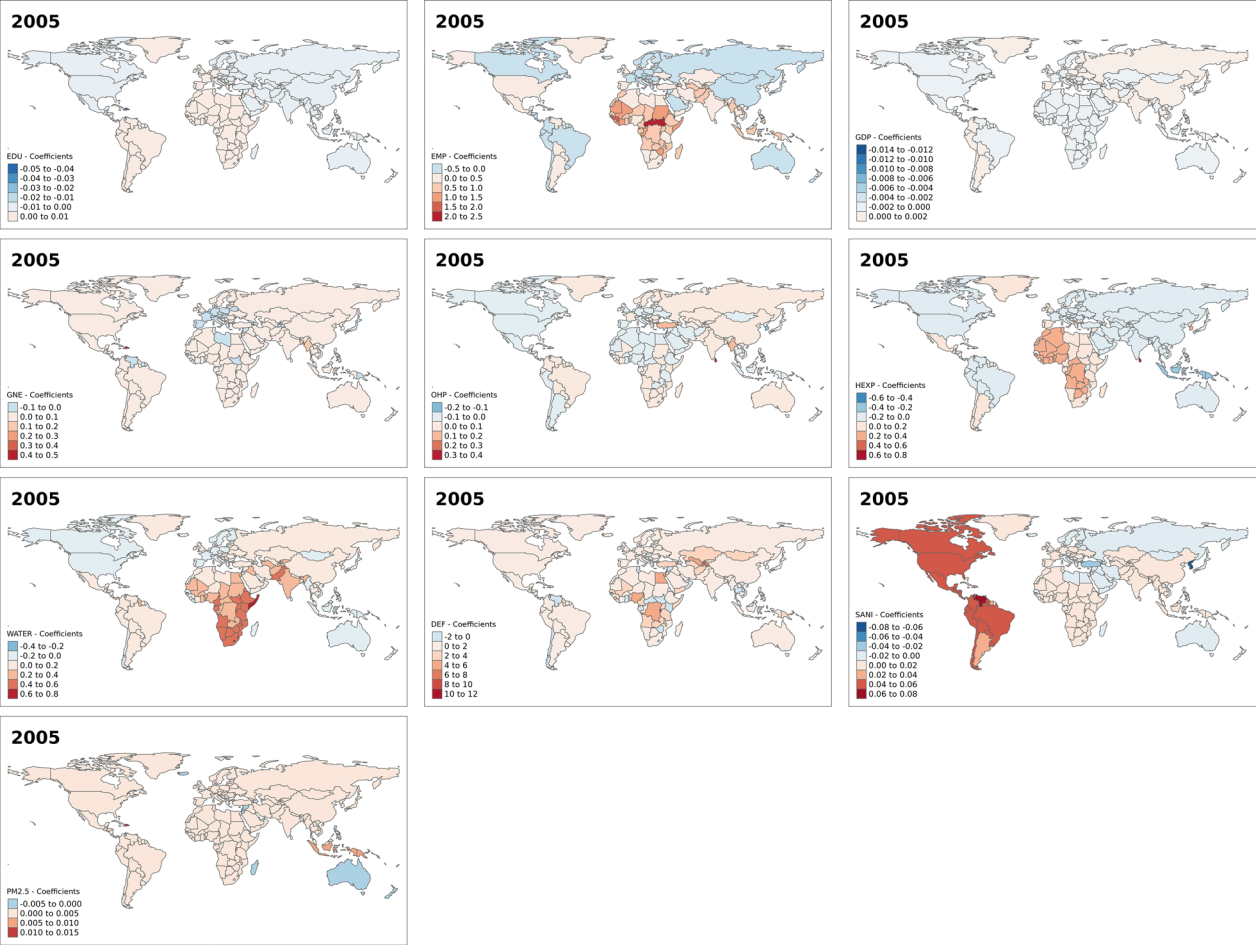
Spatial varying coefficients for the effects of development indicators on under five mortality rates in year 2005. EDU (Secondary education, pupils (% female)); EMP (Employment to population ratio, 15 + , female (%) (ILO estimate)); GDP (Gross domestic product—per capita (current US$)); GNE (Gross national expenditure (% of GDP)); OHP (Out-of-pocket expenditure (% of current health expenditure)); HEXP (Current health expenditure (% of GDP)); WATER (People using at least basic drinking water services (% of population)); DEF (People practicing open defecation (% of population)); SANI (People using at least basic sanitation services (% of population)); PM (PM2.5 air pollution, mean annual exposure (micrograms per cubic meter))

**Fig. 5:**
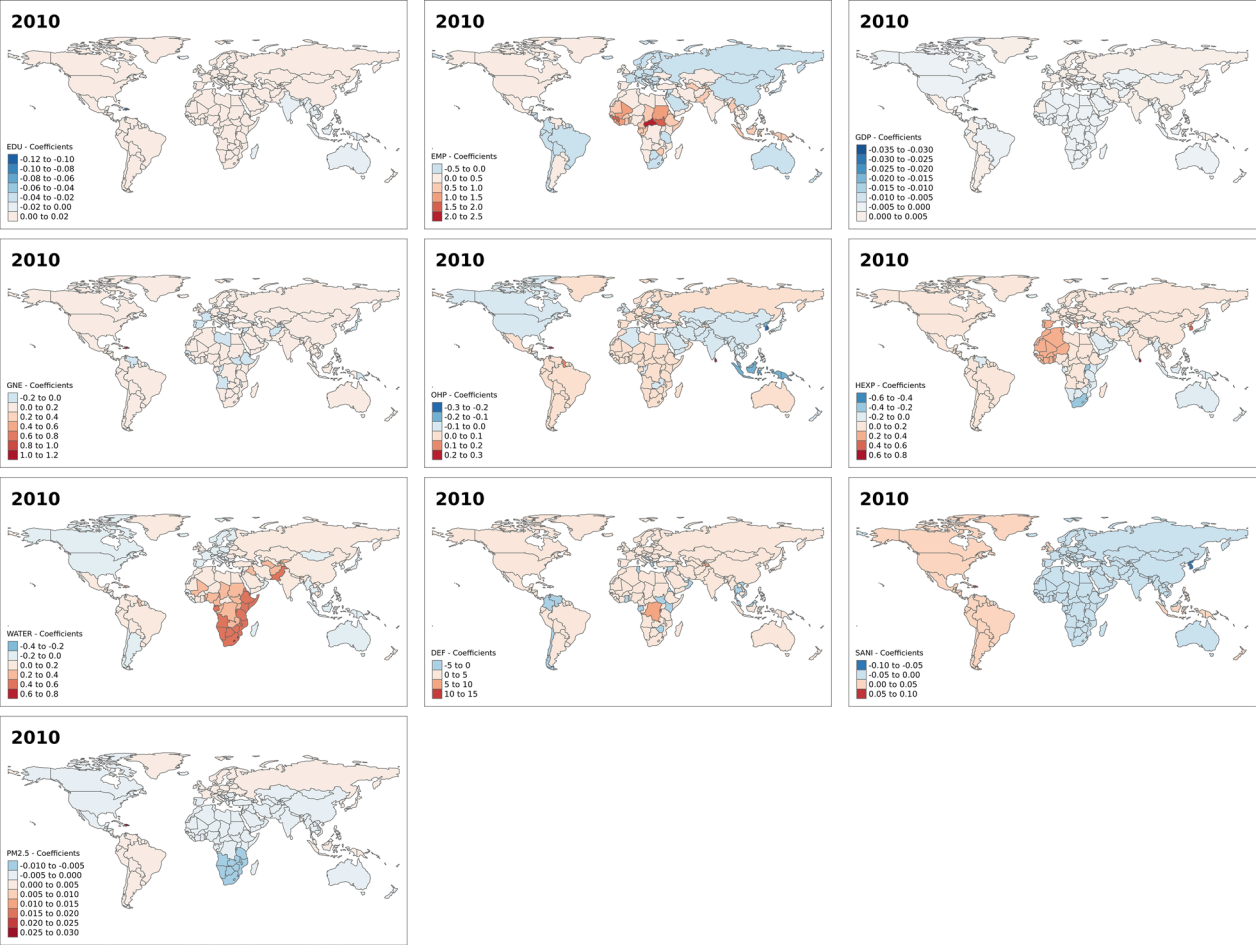
Spatial varying coefficients for the effects of development indicators on under five mortality rates in year 2010. EDU (Secondary education, pupils (% female)); EMP (Employment to population ratio, 15 + , female (%) (ILO estimate)); GDP (Gross domestic product—per capita (current US$)); GNE (Gross national expenditure (% of GDP)); OHP (Out-of-pocket expenditure (% of current health expenditure)); HEXP (Current health expenditure (% of GDP)); WATER (People using at least basic drinking water services (% of population)); DEF (People practicing open defecation (% of population)); SANI (People using at least basic sanitation services (% of population)); PM (PM2.5 air pollution, mean annual exposure (micrograms per cubic meter))

**Fig. 6:**
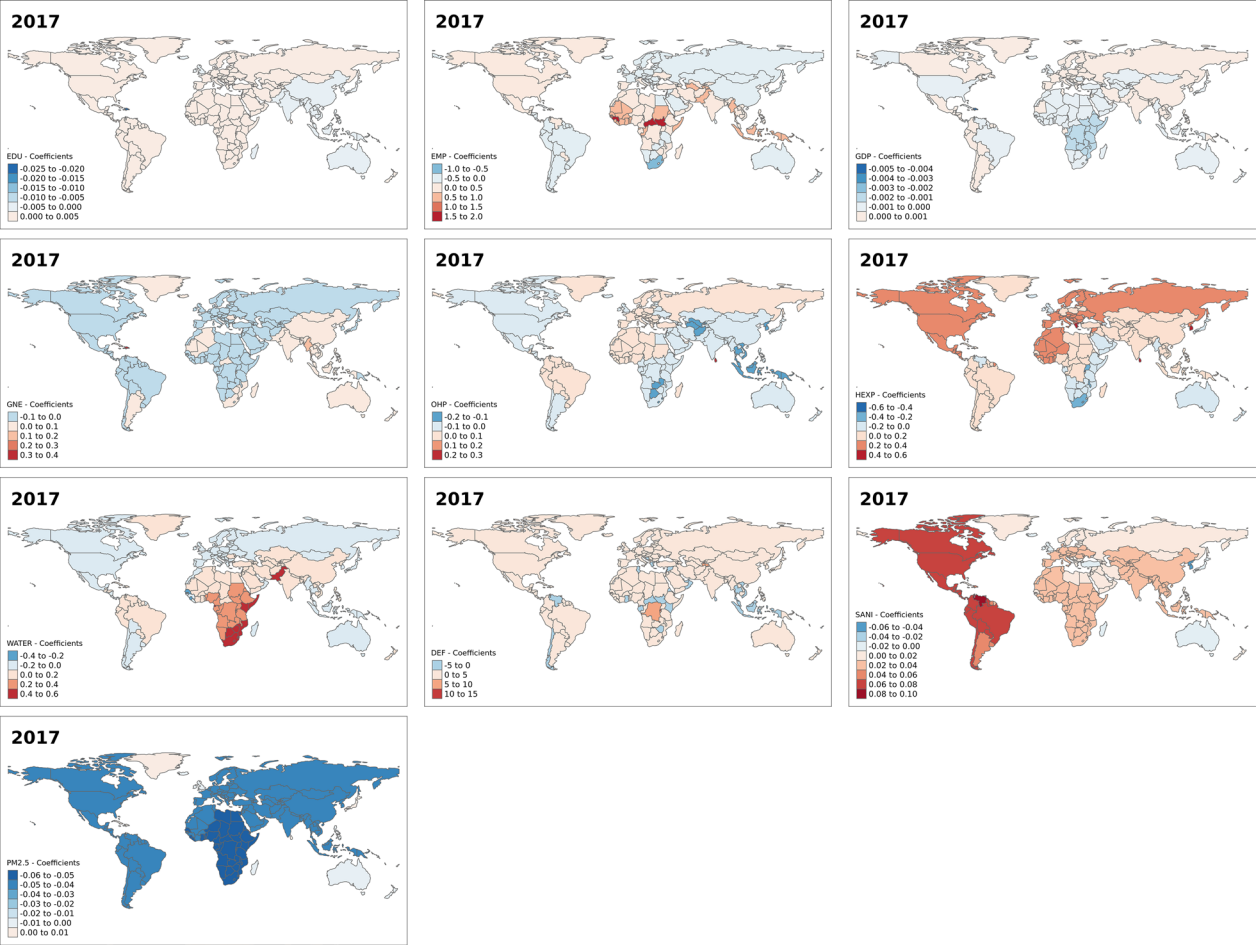
Spatial varying coefficients for the effects of development indicators on under five mortality rates in year 2017. EDU (Secondary education, pupils (% female)); EMP (Employment to population ratio, 15 + , female (%) (ILO estimate)); GDP (Gross domestic product—per capita (current US$)); GNE (Gross national expenditure (% of GDP)); OHP (Out-of-pocket expenditure (% of current health expenditure)); HEXP (Current health expenditure (% of GDP)); WATER (People using at least basic drinking water services (% of population)); DEF (People practicing open defecation (% of population)); SANI (People using at least basic sanitation services (% of population)); PM (PM2.5 air pollution, mean annual exposure (micrograms per cubic meter))

This section focuses on the results of the spatial heterogeneity or variation model—that is, in the spatial varying association between development indicators and under-five mortality rates. Figure 3 shows the results for the effect of the development indicator on under-five mortality for the year 2000. From the maps, it is evident in most countries increase in the percentage of female secondary education pupils, an increase in GDP and an increase in health expenditure reduce under-five mortality. While in most countries, an increase in the percentage of employed females, out-of-pocket expenditure, access to basic drinking water, practising open defecation and PM_2.5_ increase under-five mortality rates. Similarly, figures for the other years—2005, 2010 and 2017—show spatial variations in the effect of the development indicators on under-five mortality across the world (Figs. 4, 5, and 6). However, we observed a reverse in the spatial trend of the association between these development indicators and under-five mortality over the years. In 2005 (Fig. 4), the effect of education, GDP and health expenditure became elevated under-five mortality rates in an increased number of countries compared to the year 2000 (Fig. 3). While, the female employment, out-of-pocket expenditure, access to basic drinking water, practising open defecation and PM_2.5_ reduced effect on under mortality rates increased in several countries. In 2017 (Fig. 6), we see a complete reversal of the effect of the indicators compared to their effect in 2000. For instance, in most countries increase in PM2.5 was associated with reduced under-five mortality rates. An increase in health expenditure and female secondary education pupils in 2017 was associated with high under-five mortality rates in most countries in the world compared to the year 2000.

Additional figures (Additional file 1: Figs. S1–S4) show that the observed effects of the development indicators are significant in some countries and not significant in others. For instance, in the year 2000, the effect of the percentage of female secondary education pupils was not significant in all countries. While the effect of some development indicators, such as GDP, out-of-pocket expenditure, access to drinking water and access to basic sanitation services were significant only in a few countries. The spatial patterns for the significant associations between the development indicators and under-five mortality also change over the years for most indicators, except for the proportion of female secondary education pupils and PM_2.5_.

## Discussion

### Summary of findings

Child mortality is a major global public health issue that disproportionately affects children in less developed countries [17]. Existing studies show the association between socioeconomic indicators and under-five mortality rates [49–51]. In this study, we explore the effect of major global development indicators on under-five mortality rates. Our study also examines the effect of these indicators on the temporal trend of under-five mortality rates, as well as, spatio-temporal variations in the effect of global development indicators on under-five mortality rates. The findings show a general decline in under-five mortality rates; however, under-five mortality rates in the sub-Saharan African region are relatively higher than rates of other countries of the world. Between 2000 and 2017, the lowest under-five mortality rate declined from 3.9 per 1000 live births to 1.8 per 1000 live births. Similarly, the highest under-five mortality also dropped from 234.0 per 1000 live births in 2000 to 125.5 per 1000 live births in 2017. The Spatio-temporal regression models also show that the percentage of female secondary education pupils, GDP, access to basic drinking water and sanitation services are associated with a reduction in under-five mortality rates; while, female employment, inadequate access to toileting facilities increase under-five mortality rates. We observed mixed results for current health expenditure as a percentage of GDP. In the parametric time trend model, current health expenditure was associated with a reduction in under-five mortality rates, while it was associated with an increase in under-five mortality in the non-parametric Type-IV space-time interaction model. The finding from the spatiotemporally varying coefficient model indicates there are spatial and temporal variations in the effect of development indicators on under-five mortality rates.

### Spatio-temporal effect of development indicators

The findings of this study show that improved access to female education and basic social and infrastructural amenities can help minimize under-five mortality rates. The findings of the study show female secondary education reduce under-five mortality. Higher education, especially among females, improve socioeconomic status and fosters good health behaviours. Existing studies show that higher maternal education reduces under-five mortality rates [52]. Evidence from existing studies and literature also points to a correlation between child mortality and access to improved sanitation and drinking water [53]. As seen from the results, the burden of under-five mortality is considerably higher in the sub-Saharan African region. The population in this part of the world also struggles with access to basic social amenities, including drinking water and sanitation [54]. Lack of toileting facilities, and limited and inadequate sanitation services among the poor and vulnerable populations increase their risk of exposure through contact with disease-causing pathogens. This increases children's susceptibility to morbidity and mortality. Evidence from existing studies also shows that national indicators of socioeconomic development, including GDP, are strongly correlated with under-five mortality rates [23, 25]. For instance, a systematic review by [5] indicates that 10% growth in GDP results in a 10% reduction in the infant mortality rate. Other studies have observed a similar reduction effect of GDP on child mortality [5].

Our models reveal that space-time interactions have a confounding effect on the association between some development indicators and under-five mortalities. For instance, female education, female employment, access to drinking water and PM2.5 became associated with under-five mortality when we assume interaction between structured spatial effect and structured temporal effect—that is, neighbouring countries influence each other, and previous years influence subsequent years (Type IV interaction). This is because the space-time interaction is not orthogonal to the fixed effects; in other words, the structure of the assumed space-time interaction is correlated with the model predictors—development indicators. This suggests spatial and temporal dependence in the association between the WDIs and under-five mortality rate. That is, the result suggests the under-five mortality rate of a country is influence by neighbouring countries and by previous mortality rates. In the Type IV interaction model (Model 6) we observed an increase in the proportion of employed females is associated with increased under-five mortality. One plausible explanation for this is that females compared to their male counterparts often work in unpaid employment, and precarious jobs and earn less wage [55, 56]. Thus, gender and wage inequities in employment could account for this direct association between female employment and under-five mortalities.

The findings also show that when we assume Type IV space-time interaction, an increase in mean annual PM_2.5_ exposure is associated with a reduction in under-five mortality. The finding is not surprising; albeit, it contradicts the evidence of existing studies that show that a higher level of mean annual PM_2.5_ exposure is associated with higher under five or child mortality [7, 8, 57]. Higher spatial-level PM_2.5_ exposure measurement could be indicative of socioeconomic development [58, 59]. Existing have found a positive correlation between air pollution and indicators of socioeconomic growth, such as GDP, due to an increase in industrial, transportation and energy consumption activities leading to pollutant emissions [60]. Socioeconomic development is also associated with a higher socioeconomic standard which improves access to resources that promote quality of life among children, including access to better health care and nutritious food.

We also observed mixed results for the effect of current health expenditure as a percentage of GDP on under-five mortality. In the parametric time-trend model, an increase in the percentage of current health expenditure was associated with a reduction in under-five mortality. In the non-parametric temporal model with Type IV space-time interaction, current health expenditure as a percentage of GDP was associated with an increase in under-five mortality. These results indicate conceptions of the type of space-time interaction can influence the nature of the association between some development indicators and under-five mortality.

### Spatio-temporal variations in the association between development indicators and under-five morality

The findings for spatial effect parameters for the parametric time-trend model show that the country-specific effect on under-five mortality (Panel A Fig. 1) is higher among countries in the sub-Saharan African region compared to other regions in the world. For the effect of neighbouring countries on under-five mortality, the result shows variations in the high and low effect countries (Panel B Fig. 1). However, clusters of high neighbouring country effects are seen in Africa and southern and eastern Asia. The temporal trend result ((Panel C Fig. 1) indicates the trend in under-five mortality is less steep in the sub-Saharan African region compared to the average trend in under-five mortality. A less steep trend was also observed among some central and southern Asian countries. The temporal trend for the Type IV interaction model confirms similar results with a less steep trend in most African, eastern and central Asian countries. A less steep trend was observed in some European, south, central and North American countries in contrast to the trend observed in the parametric model. The Spatiotemporally varying coefficient model also shows variations in the association between the development indicators and under-five mortality across space and over time. This shows that the effect of development indicators on under-five mortality rates is not the same across all countries. National socioeconomic and policy contexts can potentially change how these indicators affect under-five mortality rates.

### Strength and limitations

Our study has its strengths and limitations. The strength of this study includes the use of nationally representative estimates of under-five and socioeconomic indicators data from a trusted source (The World Bank). Another key strength of this study is the use of spatiotemporal modelling to understand the spatial and temporal patterns of the associations between socioeconomic development and environmental indicators and under-five. To the best of our knowledge, this study is the first to explore spatiotemporal variations in the associations between the development indicators and under-five mortality in a global context. A major limitation of this study is the geographic unit of analysis. The use of countries as spatial units of analysis ignores spatial differences in socioeconomic development and child mortalities within these countries. However, the curated data only exist at the national level hence the authors could use lower administrative units as geographic units of analysis. Another limitation is that the data used in this study covers 18 years period and did not include data from the past five years (due to the unavailability of the data for some indicators).

## Conclusion

The findings of this study show that the development indicators of countries are associated with under-five mortality rates. From the finding, we observed that indicators of good socioeconomic development such as female education, GDP, current health expenditure access to drinking water and sanitation were associated with a reduction in under-five mortality rates. On the other hand, open defecation—an indicator of underdevelopment—was associated with higher under-five mortality rates. The findings also indicate the trend of under-five mortality rates in the sub-Saharan African region is less steep compared to the global average. Given there has been a general decline in under-five mortality rates, this result shows the trend in reduction in the sub-Saharan African region has been slower than the global trend. The burden of under-five mortality rates is considerably higher among sub-Saharan African countries and some southern Asian countries as evident in the result of the country-specific effects of under-five mortality. Investment in female education, health expenditure and access to basic social amenities (water and sanitation) through sound macroeconomic policies in these high burden countries can help reduce global under-five mortality rates.

## Supplementary Information

**Additional file 1: Table S1.** Summary information of countries included in this study. **Table S2.** Proportion of missingness and number of data points (n=3,510). **Table S3.** Diagnostics information for space-time interaction model selection. **Figure S1.** Spatial varying coefficients for the effects of development indicators on under-five mortality rates in the year 2000 showing countries with significant observed effects (95% Credible Interval). EDU (Secondary education, pupils (% female)); EMP (Employment to population ratio, 15+, female (%) (ILO estimate)); GDP (Gross domestic product—per capita (current US$)); GNE (Gross national expenditure (% of GDP)); OHP (Out-of-pocket expenditure (% of current health expenditure)); HEXP (Current health expenditure (% of GDP)); WATER (People using at least basic drinking water services (% of population)); DEF (People practicing open defecation (% of population)); SANI (People using at least basic sanitation services (% of population)); PM (PM2.5 air pollution, mean annual exposure (micrograms per cubic meter)). **Figure S2.** Spatial varying coefficients for the effects of development indicators on under-five mortality rates in the year 2005 showing countries with significant observed effects (95% Credible Interval). EDU (Secondary education, pupils (% female)); EMP (Employment to population ratio, 15+, female (%) (ILO estimate)); GDP (Gross domestic product—per capita (current US$)); GNE (Gross national expenditure (% of GDP)); OHP (Out-of-pocket expenditure (% of current health expenditure)); HEXP (Current health expenditure (% of GDP)); WATER (People using at least basic drinking water services (% of population)); DEF (People practicing open defecation (% of population)); SANI (People using at least basic sanitation services (% of population)); PM (PM2.5 air pollution, mean annual exposure (micrograms per cubic meter)). **Figure S3.** Spatial varying coefficients for the effects of development indicators on under-five mortality rates in the year 2010 showing countries with significant observed effects (95% Credible Interval). EDU (Secondary education, pupils (% female)); EMP (Employment to population ratio, 15+, female (%) (ILO estimate)); GDP (Gross domestic product—per capita (current US$)); GNE (Gross national expenditure (% of GDP)); OHP (Out-of-pocket expenditure (% of current health expenditure)); HEXP (Current health expenditure (% of GDP)); WATER (People using at least basic drinking water services (% of population)); DEF (People practicing open defecation (% of population)); SANI (People using at least basic sanitation services (% of population)); PM (PM2.5 air pollution, mean annual exposure (micrograms per cubic meter)). **Figure S4.** Spatial varying coefficients for the effects of development indicators on under-five mortality rates in the year 2017 showing countries with significant observed effects (95% Credible Interval). EDU (Secondary education, pupils (% female)); EMP (Employment to population ratio, 15+, female (%) (ILO estimate)); GDP (Gross domestic product—per capita (current US$)); GNE (Gross national expenditure (% of GDP)); OHP (Out-of-pocket expenditure (% of current health expenditure)); HEXP (Current health expenditure (% of GDP)); WATER (People using at least basic drinking water services (% of population)); DEF (People practicing open defecation (% of population)); SANI (People using at least basic sanitation services (% of population)); PM (PM2.5 air pollution, mean annual exposure (micrograms per cubic meter)). **Table S4.** Number of countries with missing information from 1960 to 2019 by study variables Number of countries with missing data for all available years by the study variables.

## Data Availability

The data used in this study are publicly available data. The world development indicator data is available via https://databank.worldbank.org/source/world-development-indicators. The data on under five mortality rate per 1000 live births is also available on https://data.worldbank.org/indicator/SH.DYN.MORT.

## References

[1] United Nations Inter-agency Group for Child Mortality Estimation (UN IGME), ‘Levels & Trends in Child Mortality: Report 2019, Estimates developed by the United Nations Inter-agency Group for Child Mortality Estimation’, United Nations Children’s Fund, New York, 2019.

[2] United Nations Department of Economic and Social Affairs. The Sustainable Development Goals Report 2019. United Nations Department of Economic and Social Affairs, New York, 2019.

[3] Sharrow D, Hug L, You D, Alkema L, Black R, Cousens S (2022). Global, regional, and national trends in under-5 mortality between 1990 and 2019 with scenario-based projections until 2030: a systematic analysis by the UN inter-agency group for child mortality estimation. Lancet Glob Heal.

[4] Chao F, You D, Pedersen J, Hug L, Alkema L (2018). National and regional under-5 mortality rate by economic status for low-income and middle-income countries: a systematic assessment. Lancet Glob Heal.

[5] O’Hare B, Makuta I, Chiwaula L, Bar-Zeev N (2013). Income and child mortality in developing countries: a systematic review and meta-analysis. J R Soc Med.

[6] Cardona M, Millward J, Gemmill A, Yoo KJ, Bishai DM (2022). Estimated impact of the 2020 economic downturn on under-5 mortality for 129 countries. PLoS ONE.

[7] Goyal N, Karra M, Canning D (2019). Early-life exposure to ambient fine particulate air pollution and infant mortality: pooled evidence from 43 low- and middle-income countries. Int J Epidemiol.

[8] Heft-Neal S, Burney J, Bendavid E, Burke M (2018). Robust relationship between air quality and infant mortality in Africa. Nature.

[9] Balaj M, York HW, Sripada K, Besnier E, Vonen HD, Aravkin A (2021). Parental education and inequalities in child mortality: a global systematic review and meta-analysis. Lancet.

[10] Hajat A, Hsia C, O’Neill MS (2015). Socioeconomic disparities and air pollution exposure: a global review. Curr Environ Heal Reports.

[11] Huang G, Zhou W, Qian Y, Fisher B (2019). Breathing the same air? Socioeconomic disparities in PM2.5 exposure and the potential benefits from air filtration. Sci Total Environ.

[12] Verhulst A, Prieto JR, Alam N, Eilerts-Spinelli H, Erchick DJ, Gerland P (2022). Divergent age patterns of under-5 mortality in south Asia and sub-Saharan Africa: a modelling study. Lancet Glob Heal.

[13] Black RE, Victora CG, Walker SP, Bhutta ZA, Christian P, De Onis M (2013). Maternal and child undernutrition and overweight in low-income and middle-income countries. Lancet.

[14] Prüss-Ustün A, Wolf J, Bartram J, Clasen T, Cumming O, Freeman MC (2019). Burden of disease from inadequate water, sanitation and hygiene for selected adverse health outcomes: an updated analysis with a focus on low- and middle-income countries. Int J Hyg Environ Health.

[15] Prüss-Ustün Annette, Bartram J, Clasen T, Colford JM, Cumming O, Curtis V (2014). Burden of disease from inadequate water, sanitation and hygiene in low- and middle-income settings: a retrospective analysis of data from 145 countries. Trop Med Int Heal.

[16] WHO (2016). Ambient air pollution: a global assessment burden of disease.

[17] Andriano L, Monden CWS (2019). The causal effect of maternal education on child mortality: evidence from a quasi-experiment in Malawi and Uganda. Demography.

[18] Mandal S, Chouhan P (2021). Maternal education and child mortality differentials: an upshot from the national family health survey 2015–2016, India. Omega (United States).

[19] Ray D, Linden M (2020). Health expenditure, longevity, and child mortality: dynamic panel data approach with global data. Int J Heal Econ Manag.

[20] Zeng W, Lannes L, Mutasa R (2018). Utilization of health care and burden of out-of-pocket health expenditure in Zimbabwe: results from a national household survey. Health Syst Reform.

[21] Shapira G, De Walque D, Friedman J (2021). How many infants may have died in low-income and middle-income countries in 2020 due to the economic contraction accompanying the COVID-19 pandemic? Mortality projections based on forecasted declines in economic growth. BMJ Open.

[22] Zakaria M, Tariq S, Husnain MI, ul (2020). Socio-economic, macroeconomic, demographic, and environmental variables as determinants of child mortality in South Asia. Environ Sci Pollut Res.

[23] Oviedo Tejada CA, Triaca LM, Liermann NH, Ewerling F, Costa JC (2019). Economic crises, child mortality and the protective role of public health expenditure. Cien Saude Colet.

[24] Pretorius CE, Asare H, Kruger HS, Genuneit J, Siziba LP, Ricci C (2021). Exclusive breastfeeding, child mortality, and economic cost in Sub-Saharan Africa. Pediatrics.

[25] Salahuddin M, Vink N, Ralph N, Gow J (2020). Effects of economic growth, foreign direct investment and internet use on child health outcomes: empirical evidence from South Africa. Routledge.

[26] Zilidis C, Hadjichristodoulou C (2020). Economic crisis impact and social determinants of perinatal outcomes and infant mortality in Greece. Int J Environ Res Public Heal.

[27] Headey D, Palloni G (2019). Water, sanitation, and child health: evidence from subnational panel data in 59 countries. Demography.

[28] Hunter PR, Prüss-Ustün A (2016). Have we substantially underestimated the impact of improved sanitation coverage on child health? A generalized additive model panel analysis of global data on child mortality and malnutrition. PLoS One.

[29] Zahid J (2018). Impact of clean drinking water and sanitation on water borne diseases in Pakistan.

[30] Alkema L, New JR (2014). Global estimation of child mortality using a Bayesian B-spline bias-reduction model. Ann Appl Stat.

[31] Pollack CE, Chideya S, Cubbin C, Williams B, Dekker M, Braveman P (2007). Should health studies measure wealth?. A systematic review. Am J Prev Med..

[32] Cole WM (2019). Wealth and health revisited: economic growth and wellbeing in developing countries, 1970 to 2015. Soc Sci Res.

[33] Cameron L, Williams J (2009). Is the relationship between socioeconomic status and health stronger for older children in developing countries?. Demography.

[34] Bloom DE, Canning D (2000). The health and wealth of nations. Science.

[35] Aber JL, Bennett NG, Conley DC, Li J (1997). The effects of poverty on child health and development. Annu Rev Public Health.

[36] Blangiardo M, Cameletti M, Baio G, Rue H (2013). Spatial and spatio-temporal models with R-INLA. Spat Spatiotemporal Epidemiol.

[37] Bernardinelli L, Clayton D, Pascutto C, Montomoli C, Ghislandi M, Songini M (1995). Bayesian analysis of space-time variation in disease risk. Stat Med.

[38] Blangiardo M, Cameletti M (2015). Spatial and spatio-temporal Bayesian Models with R-INLA.

[39] Knorr-Held L (2000). Bayesian modelling of inseparable space-time variation in disease risk. Stat Med.

[40] Song C, Wang Y, Yang X, Yang Y, Tang Z, Wang X (2020). Spatial and temporal impacts of socioeconomic and environmental factors on healthcare resources: a county-level bayesian local spatiotemporal regression modeling study of hospital beds in Southwest China. Int J Environ Res Public Health.

[41] van Niekerk J, Bakka H, Rue H, Schenk O (2021). New frontiers in Bayesian modeling using the INLA Package in R. J Stat Softw.

[42] R Core Team (2020). R: a language and environment for statistical computing.

[43] Bakka H, Fuglstad G, Riebler A, Bolin D, Krainski E, Simpson D (2018). Spatial modelling with R-INLA: a review. WIREs Comp Stat.

[44] Rue H, Martino S, Chopin N (2009). Approximate bayesian inference for latent Gaussian models by using integrated nested Laplace approximations. J R Stat Soc Ser B Stat Methodol.

[45] Martino S, Akerkar R, Rue H (2011). Approximate Bayesian Inference for Survival Models. Scand J Stat.

[46] Tennekes M (2018). tmap: thematic maps in R. J Stat Softw.

[47] Spiegelhalter DJ, Best NG, Carlin BP, Van Der Linde A (2002). Bayesian measures of model complexity and fit. J R Stat Soc Ser B Stat Methodol.

[48] Watanabe S (2010). Asymptotic equivalence of Bayes cross validation and widely applicable information criterion in singular learning theory. J Mach Learn Res.

[49] Black RE, Cousens S, Johnson HL, Lawn JE, Rudan I, Bassani DG (2010). Global, regional, and national causes of child mortality in 2008: a systematic analysis. Lancet.

[50] Mensch BS, Chuang EK, Melnikas AJ, Psaki SR (2019). Systematic review evidence for causal links between education and maternal and child health: systematic review. SSRN J.

[51] Tanaka S (2015). Environmental regulations on air pollution in China and their impact on infant mortality. J Health Econ.

[52] Gakidou E, Cowling K, Lozano R, Murray CJ (2010). Increased educational attainment and its effect on child mortality in 175 countries between 1970 and 2009: a systematic analysis. Lancet.

[53] Balakrishnan K, Dey S, Gupta T, Dhaliwal RS, Brauer M, Cohen AJ (2019). The impact of air pollution on deaths, disease burden, and life expectancy across the states of India: the global burden of disease study 2017. Lancet Planet Heal.

[54] Wolf J, Hunter PR, Freeman MC, Cumming O, Clasen T, Bartram J (2018). Impact of drinking water, sanitation and handwashing with soap on childhood diarrhoeal disease: updated meta-analysis and meta-regression. Trop Med Int Heal.

[55] Siegel PA, Hambrick DC (2005). Pay disparities within top management groups: evidence of harmful effects on performance of high-technology firms. Organ Sci.

[56] Perfect D (2011). Gender pay gaps.

[57] Son JY, Bell ML, Lee JT (2011). Survival analysis of long-term exposure to different sizes of airborne particulate matter and risk of infant mortality using a birth cohort in Seoul, Korea. Environ Health Perspect.

[58] Li Z, Chen WT, Chang IC, Hung CC (2021). Dynamic relationship between air pollution and economic growth in Taiwan deduced from mathematical models. Clean Soil Air Water.

[59] Dechezleprêtre A, Rivers N, Stadler B. The economic cost of air pollution: Evidence from Europe. 2020. Report No.: 1584.

[60] Bubalo M, van Zanten BT, Verburg PH (2019). Crowdsourcing geo-information on landscape perceptions and preferences: a review. Landsc Urban Plan.

