# Supplementary material for: Spatiotemporal analysis of the effect of global development indicators on child mortality
Source: Int J Health Geogr. 2023 May 4;22:9. doi: 10.1186/s12942-023-00330-x (PMC10157969; doi:10.1186/s12942-023-00330-x)
Supplement: Supplementary file 1 — Additional file 1: Table S1. Summary information of countries included in this study. Table S2. Proportion of missingness and number of data points (n=3,510). Table S3. Diagnostics information for space-time interaction model selection. Figure S1. Spatial varying coefficients for the effects of development indicators on under-five mortality rates in the year 2000 showing countries with significant observed effects (95% Credible Interval). EDU (Secondary education, pupils (% female)); EMP (Employment to population ratio, 15+, female (%) (ILO estimate)); GDP (Gross domestic product—per capita (current US$)); GNE (Gross national expenditure (% of GDP)); OHP (Out-of-pocket expenditure (% of current health expenditure)); HEXP (Current health expenditure (% of GDP)); WATER (People using at least basic drinking water services (% of population)); DEF (People practicing open defecation (% of population)); SANI (People using at least basic sanitation services (% of population)); PM (PM2.5 air pollution, mean annual exposure (micrograms per cubic meter)). Figure S2. Spatial varying coefficients for the effects of development indicators on under-five mortality rates in the year 2005 showing countries with significant observed effects (95% Credible Interval). EDU (Secondary education, pupils (% female)); EMP (Employment to population ratio, 15+, female (%) (ILO estimate)); GDP (Gross domestic product—per capita (current US$)); GNE (Gross national expenditure (% of GDP)); OHP (Out-of-pocket expenditure (% of current health expenditure)); HEXP (Current health expenditure (% of GDP)); WATER (People using at least basic drinking water services (% of population)); DEF (People practicing open defecation (% of population)); SANI (People using at least basic sanitation services (% of population)); PM (PM2.5 air pollution, mean annual exposure (micrograms per cubic meter)). Figure S3. Spatial varying coefficients for the effects of development indicators on under-five mortality rates in [file 12942_2023_330_MOESM1_ESM.docx]

Additional Material – Spatiotemporal analysis of the effect of global development indicators on child mortality

Prince M. Amegbor^1^ & Angelina Addae^2^

^1^ Global Environmental Public Health,

School of Global Public Health,

New York University,

708 Broadway, New York, NY 10003.

^2^ Department of Economics,

University of Saskatchewan,

129,72 Campus Drive,

Saskatoon SK S7N 5B5

Correspondence to: Prince Michael Amegbor, Global Environmental Public Health,

School of Global Public Health, New York University, 708 Broadway, New York, NY 10003. Email: [prince.amegbor@nyu.edu](mailto:prince.amegbor@nyu.edu)

# Overview of Additional information

1. Appendix A – Table S1. Summary information of countries included in this study.
2. Appendix B – Detailed description of data and research methods
   1. Supplementary Table S2. Proportion of missingness and number of data points (n=3,510)
   2. Supplementary Table S3. Diagnostics information for space-time interaction model selection.
3. Appendix C – Supplementary Figures
   1. Supplementary Figure S1.
   2. Supplementary Figure S2
   3. Supplementary Figure S3
   4. Supplementary Figure S4
4. Appendix D – Missing Information from 1960 to 2018 by Study Variables
5. Reference

# Appendix A – Table S1. Summary information of countries included in this study

| Table S1. Summary statistics of study variables by countries included in the study | | | | | | | | | | | |
| --- | --- | --- | --- | --- | --- | --- | --- | --- | --- | --- | --- |
| **Country** | U5MR | EDU | EMP | GDP | GNE | OHP | HEXP | WATER | DEF | SANI | PM |
| Afghanistan | 95.1 | 28.2 | 13.9 | 430.3 | 134.2 | 77.5 | 9.7 | 45.4 | 19.6 | 32.6 | 8.7 |
| Albania | 15.8 | 47.7 | 39.9 | 3,342.1 | 122.1 | - | - | 88.7 | 0.4 | 94.1 | 12.4 |
| Algeria | 30.4 | 50.5 | 11.2 | 3,784.2 | 91.4 | 26.9 | 4.8 | 91.9 | 3.1 | 86.0 | 1.8 |
| Andorra | 4.6 | 49.0 | - | 37,712.1 | - | 46.7 | 9.4 | 100.0 | 0.0 | 100.0 | 5.7 |
| Angola | 137.8 | 42.8 | 71.8 | 3,042.1 | 86.2 | 31.2 | 3.0 | 49.0 | 31.1 | 39.0 | 14.8 |
| Antigua and Barbuda | 10.7 | 50.6 | - | 13,145.3 | - | 26.9 | 5.0 | 97.3 | 0.7 | 85.5 | 0.4 |
| Argentina | 15.0 | 51.2 | 43.1 | 9,087.9 | 96.6 | 22.9 | 8.1 | 97.9 | 1.9 | 91.3 | 2.9 |
| Armenia | 20.7 | 48.7 | 39.5 | 2,640.7 | 125.7 | 72.8 | 7.8 | 97.7 | 0.0 | 90.3 | 13.8 |
| Australia | 5.0 | 45.4 | 54.6 | 44,026.5 | 101.1 | 19.4 | 8.4 | 99.8 | 0.0 | 100.0 | 0.5 |
| Austria | 4.5 | 47.7 | 49.8 | 42,012.8 | 96.8 | 18.8 | 9.9 | 100.0 | 0.0 | 100.0 | 8.1 |
| Azerbaijan | 44.2 | - | 55.2 | 4,016.8 | 88.0 | 80.0 | 5.3 | 84.0 | 0.2 | 79.1 | 20.5 |
| Bahamas | 13.4 | 50.4 | 60.4 | 29,560.7 | 100.8 | 27.2 | 5.3 | 98.6 | 0.6 | 91.8 | 1.8 |
| Bahrain | 9.4 | 49.6 | 39.3 | 19,772.2 | 82.5 | 27.9 | 3.9 | 100.0 | 0.0 | 100.0 | 17.2 |
| Bangladesh | 55.9 | 51.2 | 27.9 | 772.9 | 105.3 | 66.8 | 2.3 | 96.3 | 8.2 | 37.1 | 58.2 |
| Barbados | 14.6 | 49.8 | 55.9 | 14,963.5 | 106.9 | 39.1 | 6.7 | 98.4 | 0.8 | 93.1 | 0.5 |
| Belarus | 7.0 | 48.0 | 53.2 | 4,675.3 | 102.4 | 26.9 | 5.7 | 97.9 | 0.0 | 94.6 | 16.4 |
| Belgium | 4.7 | 51.9 | 42.2 | 39,282.0 | 97.5 | 18.6 | 9.5 | 100.0 | 0.0 | 99.5 | 13.1 |
| Belize | 19.1 | 50.9 | 39.5 | 4,199.1 | 107.5 | 27.4 | 5.2 | 92.9 | 2.6 | 85.2 | 3.9 |
| Benin | 115.5 | 35.9 | 66.5 | 950.6 | 104.7 | 46.9 | 4.0 | 64.1 | 60.5 | 12.7 | 9.2 |
| Bhutan | 49.6 | 49.1 | 59.7 | 1,876.5 | 123.5 | 16.2 | 3.8 | 91.0 | 4.8 | 59.8 | 12.6 |
| Bolivia | 47.5 | 48.3 | 58.3 | 1,899.2 | 98.7 | 31.4 | 5.4 | 86.5 | 23.0 | 46.1 | 10.6 |
| Bosnia and Herzegovina | 7.7 | 49.1 | 23.5 | 3,904.9 | 129.0 | 35.0 | 8.9 | 96.7 | 0.2 | 95.2 | 13.4 |
| Botswana | 60.0 | 51.1 | 45.2 | 5,848.8 | 95.9 | 6.3 | 5.8 | 81.6 | 16.4 | 64.4 | 17.1 |
| Brazil | 22.1 | 51.1 | 47.3 | 7,814.6 | 100.0 | 32.0 | 8.3 | 95.9 | 4.8 | 80.7 | 9.6 |
| Brunei Darussalam | 10.0 | 48.8 | 52.5 | 30,603.5 | 65.2 | 11.3 | 2.2 | 99.9 | 2.6 | 96.3 | 9.4 |
| Bulgaria | 11.8 | 47.8 | 42.3 | 5,591.4 | 106.6 | 43.5 | 7.2 | 99.5 | 0.0 | 85.9 | 11.7 |
| Burkina Faso | 127.5 | 42.9 | 57.8 | 564.3 | 111.2 | 33.4 | 5.1 | 51.6 | 59.0 | 15.9 | 6.0 |
| Burundi | 103.8 | 44.6 | 80.1 | 204.8 | 119.1 | 32.4 | 8.2 | 55.7 | 2.7 | 45.8 | 17.2 |
| Cote d'Ivoire | 113.8 | 41.8 | 45.4 | 1,220.5 | 93.7 | 54.5 | 5.4 | 71.9 | 30.7 | 26.0 | 10.6 |
| Cabo Verde | 25.6 | 52.8 | 41.8 | 2,772.8 | 122.3 | 25.2 | 4.6 | 82.4 | 35.2 | 57.6 | 1.6 |
| Cambodia | 55.9 | 39.8 | 76.7 | 744.8 | 106.1 | 57.9 | 6.7 | 65.3 | 58.2 | 33.9 | 15.6 |
| Cameroon | 113.8 | 44.4 | 71.1 | 1,167.3 | 103.4 | 72.4 | 4.3 | 60.0 | 7.2 | 39.3 | 15.0 |
| Canada | 5.7 | 48.5 | 57.1 | 40,428.4 | 99.1 | 14.7 | 9.8 | 99.2 | 0.0 | 99.6 | 4.4 |
| Central African Republic | 151.5 | 36.7 | 62.6 | 391.5 | 109.8 | 43.1 | 4.5 | 50.7 | 23.7 | 21.1 | 14.8 |
| Chad | 155.1 | 27.9 | 63.2 | 678.5 | 112.2 | 59.4 | 4.8 | 40.1 | 69.0 | 10.4 | 2.9 |
| Chile | 8.9 | 49.6 | 40.3 | 10,512.6 | 95.6 | 37.8 | 7.2 | 98.0 | 1.2 | 96.4 | 16.7 |
| China | 19.8 | 47.3 | 63.0 | 4,228.6 | 96.2 | 48.0 | 4.4 | 87.0 | 1.4 | 70.6 | 17.5 |
| Colombia | 19.5 | 51.2 | 46.7 | 5,079.7 | 103.3 | 15.9 | 6.6 | 94.3 | 5.8 | 80.7 | 15.1 |
| Comoros | 89.5 | 47.4 | 32.5 | 1,181.2 | 118.6 | 78.4 | 9.2 | 84.9 | 0.6 | 33.5 | 1.2 |
| Congo, Dem. Rep. | 123.5 | 36.8 | 64.0 | 334.1 | 105.9 | 51.5 | 3.8 | 38.7 | 11.3 | 20.6 | 21.6 |
| Congo, Rep. | 75.3 | 43.6 | 56.2 | 2,311.4 | 78.8 | 45.4 | 2.3 | 64.5 | 8.5 | 15.5 | 23.9 |
| Costa Rica | 10.3 | 50.2 | 41.0 | 7,377.7 | 102.3 | 27.6 | 7.4 | 97.4 | 0.5 | 95.4 | 3.0 |
| Croatia | 6.1 | 49.7 | 38.9 | 11,546.7 | 104.8 | 13.3 | 7.1 | 99.1 | 0.4 | 96.3 | 15.1 |
| Cuba | 6.6 | 48.9 | 38.7 | 5,357.3 | 98.5 | 13.1 | 9.8 | 94.3 | 1.0 | 89.7 | 3.9 |
| Cyprus | 4.2 | 49.1 | 50.6 | 25,621.3 | 101.4 | 47.8 | 6.1 | 99.8 | 0.0 | 99.6 | 7.5 |
| Czech Republic | 3.9 | 49.1 | 46.7 | 16,160.4 | 97.0 | 13.2 | 6.7 | 99.8 | 0.0 | 99.1 | 16.8 |
| Denmark | 4.6 | 49.5 | 56.3 | 51,725.8 | 94.1 | 14.3 | 9.6 | 100.0 | 0.0 | 99.6 | 7.7 |
| Djibouti | 80.6 | 41.6 | 43.4 | 1,497.0 | 103.2 | 33.2 | 4.4 | 75.5 | 18.3 | 53.6 | 7.0 |
| Dominica | 21.6 | 50.6 | - | 6,262.6 | - | 35.5 | 5.4 | 95.4 | 8.8 | 72.7 | 0.7 |
| Dominican Republic | 35.0 | 52.8 | 39.4 | 4,943.2 | 106.5 | 48.7 | 5.2 | 93.6 | 3.9 | 80.8 | 2.8 |
| Ecuador | 20.3 | 49.8 | 49.2 | 4,186.5 | 101.4 | 52.0 | 6.5 | 88.4 | 7.9 | 78.9 | 8.8 |
| Egypt, Arab Rep. | 32.1 | 48.0 | 16.5 | 2,202.7 | 105.8 | 61.8 | 4.9 | 98.5 | 0.7 | 93.2 | 6.3 |
| El Salvador | 21.8 | 49.7 | 44.2 | 2,917.9 | 119.1 | 36.7 | 8.1 | 87.7 | 5.8 | 85.3 | 8.0 |
| Equatorial Guinea | 120.1 | 36.2 | 49.7 | 12,584.5 | 69.6 | 73.1 | 2.1 | 58.6 | 5.8 | 61.9 | 13.2 |
| Eritrea | 60.8 | 41.6 | 67.2 | 424.5 | 142.2 | 48.5 | 4.1 | 49.3 | 73.5 | 10.5 | 6.0 |
| Estonia | 5.8 | 49.1 | 49.8 | 13,819.8 | 101.1 | 21.8 | 5.6 | 99.5 | 0.0 | 99.4 | 8.8 |
| Eswatini | 98.9 | 49.5 | 32.7 | 3,227.6 | 104.7 | 12.6 | 7.2 | 61.1 | 14.9 | 54.5 | 15.2 |
| Ethiopia | 94.1 | 41.3 | 71.0 | 348.7 | 116.9 | 37.8 | 4.4 | 30.6 | 50.5 | 5.4 | 16.0 |
| Fiji | 23.6 | 50.6 | 37.1 | 3,986.9 | - | 17.6 | 3.5 | 94.2 | 0.4 | 87.3 | 0.2 |
| Finland | 3.2 | 50.7 | 51.4 | 41,913.7 | 96.8 | 20.3 | 8.4 | 100.0 | 0.0 | 99.4 | 6.3 |
| France | 4.5 | 49.0 | 45.4 | 36,405.7 | 100.2 | 9.0 | 10.7 | 100.0 | 0.0 | 98.7 | 9.8 |
| Gabon | 66.3 | - | 29.4 | 7,438.8 | 73.3 | 40.3 | 3.0 | 83.2 | 2.4 | 41.8 | 16.9 |
| Gambia, The | 83.9 | 48.7 | 43.0 | 670.1 | 110.9 | 23.7 | 3.2 | 76.4 | 3.1 | 46.7 | 4.6 |
| Georgia | 19.3 | 48.4 | 48.7 | 2,812.0 | 119.8 | 71.4 | 8.3 | 95.5 | 0.5 | 92.3 | 10.1 |
| Germany | 4.4 | 47.8 | 48.5 | 38,331.6 | 94.8 | 13.4 | 10.5 | 100.0 | 0.0 | 99.2 | 12.3 |
| Ghana | 74.1 | 45.9 | 63.3 | 1,158.5 | 114.7 | 42.2 | 3.8 | 72.9 | 20.0 | 13.2 | 12.3 |
| Greece | 4.6 | 48.0 | 34.5 | 21,718.9 | 107.3 | 33.7 | 8.6 | 99.9 | 0.4 | 98.3 | 9.2 |
| Greenland | - | - | - | 38,533.5 | 120.1 | - | - | 100.0 | 0.0 | 99.6 | 1.0 |
| Grenada | 14.7 | 49.7 | - | 7,189.3 | - | 51.2 | 5.5 | 95.0 | 2.9 | 91.3 | 0.5 |
| Guatemala | 37.9 | 47.6 | 39.8 | 2,810.0 | 112.4 | 58.8 | 6.2 | 90.1 | 9.3 | 64.4 | 9.1 |
| Guinea | 126.4 | 33.2 | 60.4 | 590.2 | 116.3 | 58.1 | 3.7 | 62.8 | 20.8 | 16.1 | 6.3 |
| Guinea-Bissau | 125.6 | 35.5 | 63.5 | 522.1 | 111.1 | 47.7 | 7.3 | 60.4 | 25.8 | 16.1 | 6.0 |
| Guyana | 38.7 | 50.0 | 32.8 | 3,580.7 | 117.6 | 37.5 | 4.8 | 91.9 | 0.9 | 82.6 | 1.6 |
| Haiti | 90.5 | - | 50.0 | 1,043.6 | 131.6 | 39.1 | 7.1 | 61.3 | 27.7 | 25.7 | 3.1 |
| Honduras | 26.1 | 53.9 | 41.1 | 1,728.8 | 118.4 | 48.3 | 7.8 | 90.0 | 12.6 | 72.2 | 6.3 |
| Hungary | 6.8 | 48.7 | 40.5 | 11,631.9 | 97.7 | 27.3 | 7.3 | 100.0 | 0.0 | 98.0 | 16.3 |
| Iceland | 2.9 | 49.6 | 69.0 | 49,201.0 | 99.5 | 17.6 | 8.7 | 100.0 | 0.0 | 98.8 | 1.2 |
| India | 63.7 | 44.5 | 25.3 | 1,099.6 | 103.8 | 68.6 | 3.7 | 85.7 | 49.0 | 37.8 | 33.6 |
| Indonesia | 37.6 | 49.0 | 46.2 | 2,338.5 | 98.2 | 46.8 | 2.7 | 82.9 | 20.7 | 57.1 | 17.0 |
| Iran, Islamic Rep. | 22.6 | 47.8 | 13.0 | 4,635.8 | 95.4 | 52.6 | 6.1 | 95.1 | 0.6 | 87.9 | 7.1 |
| Iraq | 36.1 | 38.9 | 10.3 | 4,548.9 | 80.0 | 44.5 | 3.1 | 88.6 | 2.2 | 80.8 | 9.4 |
| Ireland | 4.8 | 50.1 | 50.4 | 50,402.2 | 84.9 | 12.8 | 8.3 | 97.3 | 0.0 | 90.4 | 4.2 |
| Israel | 5.0 | 48.9 | 51.3 | 28,209.8 | 99.7 | 25.7 | 7.1 | 100.0 | 0.0 | 100.0 | 8.0 |
| Italy | 4.2 | 48.2 | 33.9 | 32,060.6 | 99.2 | 22.5 | 8.5 | 99.7 | 0.0 | 98.8 | 9.7 |
| Jamaica | 18.7 | 50.4 | 47.0 | 4,424.2 | 118.4 | 25.5 | 5.2 | 91.9 | 0.8 | 84.5 | 2.3 |
| Japan | 3.4 | 48.8 | 47.0 | 38,711.2 | 99.5 | 14.7 | 9.0 | 98.7 | 0.0 | 100.0 | 9.3 |
| Jordan | 21.5 | 49.7 | 10.6 | 3,099.5 | 125.3 | 31.5 | 8.5 | 99.2 | 0.2 | 98.0 | 5.8 |
| Kazakhstan | 24.4 | 49.4 | 59.6 | 7,116.4 | 88.7 | 33.8 | 3.3 | 90.1 | 0.0 | 97.3 | 9.1 |
| Kenya | 67.7 | 48.2 | 64.5 | 877.8 | 112.2 | 35.6 | 5.4 | 53.3 | 13.8 | 31.3 | 7.6 |
| Kiribati | 63.9 | 52.8 | - | 1,342.8 | 179.7 | 0.1 | 10.7 | 60.8 | 38.6 | 36.4 | 12.8 |
| Korea, Dem. People's Rep. | 32.3 | 49.1 | 72.6 | - | - | - | - | 97.4 | 0.0 | 78.2 | 16.4 |
| Korea, Rep. | 4.8 | 47.4 | 48.7 | 21,866.6 | 97.1 | 36.5 | 5.8 | 99.0 | 0.0 | 100.0 | 19.9 |
| Kosovo | - | - | - | 2,848.7 | 133.9 | - | - | - | - | - | 12.2 |
| Kuwait | 10.5 | 49.6 | 43.8 | 35,509.2 | 72.2 | 16.1 | 3.1 | 100.0 | 0.0 | 100.0 | 16.5 |
| Kyrgyzstan | 33.2 | 49.5 | 47.3 | 807.3 | 127.3 | 48.2 | 6.6 | 84.1 | 0.1 | 94.8 | 6.7 |
| Lao PDR | 75.0 | 44.2 | 76.7 | 1,120.6 | 113.5 | 44.1 | 3.1 | 64.8 | 40.4 | 52.1 | 33.8 |
| Latvia | 8.7 | 48.8 | 46.9 | 10,913.9 | 107.9 | 41.0 | 5.8 | 98.2 | 0.0 | 90.1 | 10.8 |
| Lebanon | 12.2 | 51.6 | 19.8 | 6,258.7 | 124.9 | 43.2 | 8.3 | 89.2 | 0.1 | 85.5 | 7.6 |
| Lesotho | 107.6 | 56.9 | 41.0 | 911.2 | 154.4 | 24.1 | 7.2 | 67.9 | 36.5 | 26.6 | 11.6 |
| Liberia | 113.9 | 43.0 | 68.6 | 486.7 | 175.0 | 57.1 | 7.5 | 68.2 | 47.3 | 15.5 | 8.9 |
| Libya | 19.4 | 51.8 | 24.3 | 7,924.9 | 64.4 | 35.9 | 3.7 | 91.8 | 0.0 | 98.8 | 2.6 |
| Liechtenstein | - | 45.4 | - | 130,274.8 | - | - | - | 100.0 | 0.0 | 100.0 | 8.4 |
| Lithuania | 7.2 | 48.3 | 47.7 | 11,027.0 | 104.0 | 29.7 | 6.2 | 93.6 | 0.0 | 88.8 | 13.7 |
| Luxembourg | 3.4 | 49.8 | 45.1 | 92,097.5 | 69.9 | 11.7 | 6.4 | 100.0 | 0.0 | 97.7 | 11.9 |
| Madagascar | 75.0 | 49.1 | 81.9 | 430.7 | 108.8 | 31.9 | 5.4 | 44.6 | 41.4 | 7.4 | 4.6 |
| Malawi | 99.5 | 46.0 | 68.8 | 338.3 | 111.6 | 11.2 | 7.3 | 60.9 | 10.6 | 23.5 | 10.9 |
| Malaysia | 8.3 | 50.7 | 44.3 | 7,717.9 | 84.4 | 35.9 | 3.3 | 97.0 | 0.8 | 98.5 | 4.9 |
| Maldives | 18.8 | 51.7 | 40.3 | 6,049.9 | - | 41.0 | 8.6 | 96.3 | 5.8 | 88.2 | 1.6 |
| Mali | 141.0 | 39.6 | 54.7 | 616.0 | 109.8 | 52.1 | 4.7 | 63.6 | 13.9 | 27.1 | 2.3 |
| Malta | 7.0 | 48.3 | 32.3 | 19,667.6 | 97.9 | 31.6 | 8.4 | 100.0 | 0.0 | 100.0 | 5.1 |
| Marshall Islands | 38.6 | 49.8 | - | 2,861.3 | 155.9 | 12.4 | 16.9 | 80.9 | 10.9 | 81.4 | 0.1 |
| Mauritania | 99.4 | 45.6 | 25.1 | 1,308.9 | 112.3 | 61.7 | 4.2 | 55.3 | 43.5 | 30.8 | 1.6 |
| Mauritius | 15.5 | 49.9 | 36.9 | 7,255.3 | 105.7 | 50.4 | 4.2 | 99.6 | 0.1 | 93.4 | 0.3 |
| Mexico | 18.8 | 51.1 | 40.3 | 8,938.0 | 100.7 | 47.8 | 5.6 | 94.6 | 5.4 | 83.4 | 6.8 |
| Micronesia, Fed. Sts. | 42.2 | 49.0 | - | 2,662.5 | - | 3.7 | 11.7 | 82.6 | 9.5 | 60.0 | 0.1 |
| Moldova | 19.6 | 49.5 | 40.7 | 1,947.7 | 133.5 | 44.7 | 8.0 | 86.0 | 0.1 | 75.2 | 15.9 |
| Monaco | 4.2 | 48.8 | - | 144,741.1 | - | 6.2 | 2.0 | 100.0 | 0.0 | 100.0 | 12.5 |
| Mongolia | 34.0 | 52.9 | 50.7 | 2,297.6 | 109.8 | 28.0 | 4.1 | 73.8 | 12.8 | 53.9 | 3.6 |
| Montenegro | 8.0 | 48.8 | 32.0 | 5,360.0 | 125.1 | - | - | 96.2 | 0.1 | 94.7 | 9.3 |
| Morocco | 35.0 | 45.0 | 22.1 | 2,452.3 | 109.0 | 54.9 | 5.1 | 75.0 | 14.1 | 78.3 | 3.4 |
| Mozambique | 116.1 | 43.7 | 81.1 | 477.1 | 124.6 | 9.8 | 5.2 | 37.1 | 43.4 | 19.4 | 6.4 |
| Myanmar | 68.8 | 49.9 | 53.9 | 711.3 | 99.8 | 78.7 | 2.6 | 64.9 | 9.4 | 67.6 | 18.2 |
| Namibia | 58.9 | 53.1 | 40.4 | 4,147.3 | 112.4 | 7.9 | 9.7 | 79.6 | 52.3 | 31.4 | 5.8 |
| Nauru | 38.0 | 50.9 | - | 7,727.5 | - | 2.6 | 13.0 | 97.8 | 1.9 | 65.6 | 0.3 |
| Nepal | 53.0 | 46.7 | 79.5 | 510.7 | 120.8 | 55.7 | 4.9 | 84.6 | 43.8 | 37.4 | 33.2 |
| Netherlands | 4.9 | 48.5 | 53.7 | 44,836.1 | 91.6 | 10.1 | 9.5 | 100.0 | 0.0 | 97.9 | 14.6 |
| New Zealand | 6.4 | 50.2 | 57.4 | 31,076.1 | 98.8 | 13.6 | 8.8 | 100.0 | 0.0 | 100.0 | 0.8 |
| Nicaragua | 24.1 | 52.8 | 42.7 | 1,487.3 | 120.5 | 39.6 | 6.9 | 81.3 | 10.9 | 66.8 | 3.4 |
| Niger | 144.5 | 39.6 | 65.9 | 406.5 | 112.1 | 57.3 | 7.1 | 43.3 | 75.8 | 9.7 | 2.8 |
| Nigeria | 145.9 | 45.1 | 51.1 | 1,835.0 | 92.7 | 70.5 | 3.7 | 59.7 | 23.2 | 33.7 | 9.5 |
| Northern Macedonia | 12.5 | 48.0 | 29.1 | 3,940.2 | 117.7 | 37.5 | 7.2 | 96.4 | 0.4 | 91.4 | 11.1 |
| Norway | 3.6 | 48.4 | 59.4 | 74,476.7 | 88.0 | 15.7 | 8.9 | 100.0 | 0.0 | 98.1 | 2.8 |
| Oman | 12.6 | 48.4 | 23.9 | 15,257.6 | 81.4 | 9.7 | 3.0 | 83.3 | 2.1 | 95.2 | 8.0 |
| Pakistan | 91.4 | 42.0 | 19.7 | 965.9 | 105.1 | 66.6 | 2.7 | 88.6 | 25.1 | 46.2 | 18.0 |
| Palau | 23.9 | 49.2 | - | 11,067.4 | 131.5 | 15.9 | 10.3 | 95.2 | 0.0 | 99.7 | 0.4 |
| Panama | 20.8 | 50.7 | 45.8 | 8,234.8 | 106.1 | 29.8 | 6.9 | 92.5 | 5.9 | 72.2 | 1.4 |
| Papua New Guinea | 60.5 | 41.1 | 53.6 | 1,677.0 | 87.7 | 9.2 | 2.4 | 36.8 | 13.7 | 17.8 | 0.9 |
| Paraguay | 27.2 | 50.1 | 51.2 | 3,646.1 | 93.7 | 46.8 | 5.2 | 88.1 | 0.8 | 80.4 | 9.6 |
| Peru | 23.5 | 48.5 | 64.3 | 4,357.9 | 97.9 | 35.7 | 4.8 | 86.2 | 12.1 | 69.6 | 6.2 |
| Philippines   Philippines | 33.0 | 51.1 | 46.2 | 2,000.0 | 102.5 | 52.8 | 3.9 | 89.5 | 8.2 | 68.0 | 8.5 |
| Poland | 6.6 | 48.3 | 42.1 | 10,409.1 | 101.2 | 25.8 | 6.1 | 98.0 | 0.0 | 93.3 | 23.5 |
| Portugal | 4.6 | 49.9 | 49.1 | 19,436.7 | 105.1 | 25.5 | 9.1 | 99.4 | 0.0 | 98.5 | 6.9 |
| Qatar | 9.6 | 49.1 | 48.9 | 59,122.9 | 67.8 | 16.6 | 2.3 | 99.9 | 0.0 | 100.0 | 15.6 |
| Romania | 14.2 | 48.8 | 45.0 | 6,902.0 | 106.3 | 20.0 | 5.0 | 100.0 | 0.0 | 78.6 | 15.0 |
| Russian Federation | 12.2 | 48.4 | 51.7 | 8,560.3 | 90.9 | 34.1 | 5.1 | 96.0 | 0.0 | 86.9 | 6.8 |
| Rwanda | 88.7 | 50.1 | 83.3 | 514.4 | 114.3 | 16.0 | 7.3 | 51.7 | 3.2 | 56.3 | 20.6 |
| Saint Kitts and Nevis | 16.3 | 51.0 | - | 14,513.6 | 100.0 | 52.1 | 4.9 | 98.7 | 1.5 | 89.4 | 0.5 |
| Saint Lucia | 18.1 | 52.0 | 44.3 | 8,139.0 | - | 54.9 | 5.5 | 93.6 | 5.6 | 85.3 | 0.7 |
| Saint Vincent and Grenadines | 20.8 | 50.9 | 43.5 | 5,794.6 | - | 32.3 | 4.3 | 94.3 | 3.1 | 80.4 | 0.6 |
| Samoa | 19.0 | 50.7 | 29.5 | 3,111.3 | - | 12.7 | 5.1 | 94.0 | 0.1 | 97.8 | 0.1 |
| San Marino | 3.5 | 48.6 | - | 54,035.5 | 74.3 | 20.8 | 6.1 | 100.0 | 0.0 | 100.0 | 13.4 |
| Sao Tome and Principe | 53.1 | 52.0 | 31.2 | 1,144.0 | - | 25.0 | 8.4 | 75.2 | 58.6 | 32.0 | 9.0 |
| Saudi Arabia | 13.9 | 47.3 | 15.6 | 17,116.8 | 81.8 | 16.8 | 4.2 | 98.6 | 0.0 | 99.1 | 11.4 |
| Senegal | 79.5 | 44.8 | 30.5 | 1,124.1 | 114.2 | 49.7 | 4.0 | 70.2 | 18.8 | 45.3 | 4.0 |
| Serbia | 8.4 | 49.3 | 36.3 | 4,901.0 | 113.4 | 33.9 | 8.6 | 90.9 | 0.1 | 96.3 | 14.6 |
| Seychelles | 14.1 | 50.4 | - | 11,586.3 | 110.3 | 27.0 | 4.8 | 95.2 | 0.7 | 97.6 | 0.5 |
| Sierra Leone | 173.8 | 45.9 | 58.7 | 409.2 | 120.5 | 63.4 | 12.5 | 50.0 | 21.9 | 12.8 | 7.9 |
| Singapore | 3.0 | 48.3 | 54.1 | 41,430.1 | 76.0 | 43.3 | 3.5 | 100.0 | 0.0 | 100.0 | 15.9 |
| Slovak Republic | 7.5 | 49.1 | 43.9 | 14,060.7 | 100.0 | 19.6 | 6.8 | 98.6 | 0.0 | 97.9 | 15.9 |
| Slovenia | 3.6 | 48.7 | 48.4 | 20,183.1 | 98.1 | 12.6 | 8.2 | 99.6 | 0.0 | 99.1 | 12.2 |
| Solomon Islands | 26.2 | 44.0 | 81.4 | 1,536.8 | 126.5 | 4.2 | 7.2 | 74.4 | 55.5 | 25.9 | 0.3 |
| Somalia | 157.2 | 31.5 | 18.6 | - | - | - | - | 36.7 | 43.0 | 27.8 | 3.1 |
| South Africa | 59.3 | 51.3 | 32.0 | 5,485.2 | 99.5 | 10.5 | 7.3 | 88.8 | 6.6 | 67.5 | 4.0 |
| South Sudan | 126.3 | 34.0 | 61.0 | 1,432.6 | 83.2 | 19.2 | 9.8 | 41.0 | 67.0 | 8.9 | 10.0 |
| Spain | 4.2 | 49.5 | 39.5 | 26,530.8 | 100.9 | 22.7 | 8.2 | 100.0 | 0.0 | 99.9 | 9.0 |
| Sri Lanka | 13.1 | 50.6 | 32.7 | 2,313.8 | 108.7 | 46.3 | 3.9 | 84.5 | 2.2 | 90.1 | 5.8 |
| Sudan | 80.9 | 47.4 | 21.4 | 1,072.3 | 103.9 | 62.7 | 5.2 | 50.5 | 39.9 | 27.1 | 3.1 |
| Suriname | 26.3 | 55.7 | 32.2 | 5,819.7 | 93.3 | 23.4 | 5.4 | 92.4 | 5.6 | 81.8 | 0.6 |
| Sweden | 3.3 | 50.5 | 55.5 | 47,992.1 | 95.0 | 16.2 | 9.3 | 100.0 | 0.0 | 99.3 | 5.0 |
| Switzerland | 4.8 | 47.6 | 57.8 | 66,594.2 | 90.8 | 30.0 | 10.9 | 100.0 | 0.0 | 99.9 | 9.7 |
| Syrian Arab Republic | 18.5 | 47.8 | 12.1 | 1,464.1 | 95.8 | 54.0 | 4.0 | 96.1 | 1.1 | 92.5 | 11.0 |
| Tajikistan | 51.3 | 45.4 | 27.0 | 609.6 | 129.2 | 68.6 | 5.7 | 68.3 | 0.6 | 93.2 | 26.2 |
| Tanzania | 83.3 | 48.6 | 80.9 | 685.9 | 105.6 | 31.2 | 5.0 | 41.4 | 10.9 | 17.1 | 9.2 |
| Thailand | 14.8 | 50.1 | 62.9 | 4,267.9 | 93.2 | 19.0 | 3.4 | 97.0 | 0.6 | 95.3 | 33.7 |
| Timor-Leste | 71.5 | 49.7 | 59.6 | 796.3 | 201.9 | 16.2 | 2.0 | 63.6 | 30.8 | 45.3 | 2.6 |
| Togo | 94.6 | 34.2 | 76.3 | 490.3 | 113.3 | 62.7 | 4.9 | 55.6 | 53.3 | 12.8 | 11.3 |
| Tonga | 17.0 | 48.8 | 44.0 | 3,262.6 | 140.0 | 12.9 | 4.4 | 99.2 | 0.2 | 91.0 | 0.2 |
| Trinidad and Tobago | 24.3 | 51.1 | 49.1 | 14,678.4 | - | 47.0 | 4.9 | 95.5 | 0.2 | 92.1 | 0.4 |
| Tunisia | 20.8 | 50.5 | 19.4 | 3,532.4 | 105.8 | 40.8 | 6.0 | 92.0 | 4.7 | 84.6 | 3.9 |
| Turkey | 21.3 | 45.6 | 24.4 | 8,813.8 | 103.2 | 19.5 | 4.8 | 97.4 | 0.4 | 90.3 | 10.9 |
| Turkmenistan | 63.4 | 48.4 | 49.0 | 3,941.4 | 78.9 | 68.9 | 6.7 | 93.3 | 0.2 | 95.2 | 8.5 |
| Tuvalu | 32.9 | 51.3 | - | 2,677.8 | - | 0.8 | 15.4 | 98.5 | 5.2 | 82.2 | 0.1 |
| Uganda | 90.9 | 44.7 | 63.5 | 561.1 | 109.8 | 37.2 | 8.8 | 37.5 | 10.0 | 18.1 | 13.0 |
| Ukraine | 12.9 | 48.3 | 44.8 | 2,378.3 | 103.7 | 44.4 | 6.4 | 95.8 | 0.0 | 95.4 | 14.7 |
| United Arab Emirates | 9.1 | 47.6 | 40.0 | 37,958.7 | 81.3 | 22.0 | 3.1 | 96.1 | 0.1 | 98.6 | 13.9 |
| United Kingdom | 5.4 | 49.3 | 52.4 | 40,281.4 | 101.8 | 11.8 | 8.0 | 100.0 | 0.0 | 99.1 | 11.9 |
| United States | 7.5 | 49.0 | 54.2 | 47,754.1 | 103.9 | 12.9 | 15.4 | 99.2 | 0.0 | 100.0 | 7.1 |
| Uruguay | 12.2 | 52.0 | 47.3 | 10,289.4 | 100.4 | 20.9 | 9.1 | 98.1 | 1.1 | 95.0 | 3.0 |
| Uzbekistan | 41.1 | 48.6 | 48.3 | 1,337.1 | 97.4 | 50.5 | 5.6 | 93.6 | 0.0 | 97.3 | 8.2 |
| Vanuatu | 28.8 | 47.3 | 57.5 | 2,422.1 | 107.6 | 9.0 | 3.5 | 85.7 | 1.6 | 50.9 | 0.5 |
| Venezuela, RB | 19.1 | 51.7 | 43.7 | 8,759.7 | 92.5 | 36.5 | 6.4 | 96.2 | 5.3 | 93.7 | 3.0 |
| Vietnam | 24.3 | - | 70.8 | 1,229.7 | 104.5 | 38.8 | 5.5 | 87.4 | 9.8 | 68.1 | 9.5 |
| Yemen, Rep. | 66.3 | 35.2 | 9.9 | 1,070.5 | - | 65.1 | 5.2 | 50.9 | 24.7 | 50.9 | 8.7 |
| Zambia | 96.9 | - | 63.7 | 1,124.1 | 100.1 | 22.8 | 5.2 | 54.8 | 21.8 | 25.0 | 14.8 |
| Zimbabwe | 84.2 | 47.9 | 72.7 | 876.5 | 112.5 | 27.8 | 7.6 | 68.2 | 27.6 | 41.9 | 11.1 |
| ***Abbreviations***: EDU = Secondary education, pupils (% female)  EMP = Employment to population ratio, 15+, female (%) (ILO estimate)  GDP = Gross domestic product - per capita (current US$) GNE = Gross national expenditure (% of GDP)  OHP = Out-of-pocket expenditure (% of current health expenditure)  HEXP = Current health expenditure (% of GDP)  WATER = People using at least basic drinking water services (% of population)  DEF = People practicing open defecation (% of population)  SANI = People using at least basic sanitation services (% of population)  PM = PM2.5 air pollution, mean annual exposure (micrograms per cubic meter) | | | | | | | | | | | |

# Appendix B – Detailed description of data and research methods

# Data and Methods

The data from this study was derived from the World Bank’s World Development Indicators (WDI). The WDI data contains statistical information on major global development indicators compiled from officially-recognised international sources, including national statistics bureaus. The WDI data is available at national, regional and global levels. Given the focus of our study, we used the national estimates from 195 countries. The original data had information on 217 countries and territories. In this study we excluded territories of sovereign states; that is, data on territories such as the Faroe Islands and the British Virgin Islands were excluded from the study. The WDI data used in this study span from 2000 to 2017.

The outcome variable of interest in this study was under-five mortality rate per 1,000 live births. The national estimates for under-five mortality rates were compiled by the UN Inter-agency Group for Child Mortality Estimation (UN IGME) which consists of the following international organisation: the United Nations Children's Fund (UNICEF), the World Health Organization (WHO), the World Bank and the Population Division of the United Nations’ Department of Economic and Social Affairs (UN DESA Population). The UN IGME under-five mortality rate estimates are based on nationally representative data, including data sourced from household surveys and censuses. To ensure quality data (estimates), the UN IGME excludes data sources with substantial non-sampling errors or omissions from the statistical model used to derive the estimates. The UN IGME estimation process does not use covariates and a detailed description of the modelling process can be found elsewhere [1].

Evidence from existing studies highlight correlation between national socioeconomic development and child health [2–4]. The research shows that contextual socioeconomic, environmental and political factors have significant impact on the health and wellbeing of children [4–6]. In view of this, we used socioeconomic and environmental indicators from the World Bank’s WDI as predictors of under-five mortality rate per 1,000 live births. The WDI has 1,442 indictors or measures grouped under 12 major domains (Economic Policy and Debt, Education, Environment, Health, Gender, Financial Sector, Infrastructure, Poverty, Private Sector and Trade, Social, Public Sectors, Social Protection and Labor). Based on the evidence from existing studies, we used nine major global development indicators as determinants or predictors of under-five mortality rate per 1,000 live births. The covariates used in this study were:

- Percentage of females with secondary education
- Percentage of females aged 15 years and above employed as a ratio of the total population
- Gross domestic product - GDP - per capita (current US$)
- Gross national expenditure as a percentage of GDP
- Percentage of out-of-pocket expenditure out of current health expenditure
- Percentage of people using at least basic drinking water services
- Percentage of the population practising open defecation
- Percentage of population using at least basic sanitation services
- Mean annual PM2.5 exposure in micrograms per cubic meter

## Analysis

## Spatio-temporal analysis

First, we examined the association between the world development indicators used in this study and under-five mortality rate per 1,000 live births. Considering the temporal dimension of the data, we employed Bayesian Spatio-temporal modelling to assess the effect of the development indicators on under-five mortality. Several space-time interaction Bayesian models for disease risk and mapping have been proposed in existing literature [7]. These models can be broadly classified into the parametric and non-parametric trends for the temporal component. These models conceptualise the nature of interaction between space and time. We employed these models to assess the best space-time interaction models in relations to the study objectives. The model for the parametric trend for the temporal component (Model 1) was proposed by Bernardinelli et al. (1995). The proposed model encapsulates the main spatial effects for the entire time period and a linear time trend. Considering our outcome (under-five mortality rate), Bernardinelli et al. (1995) proposed model can be expressed as:

$\eta_{it}=b_{0}+u_{i}+v_{i}+\left( \beta+\delta_{i} \right)\times t$ (1)

$$y_{it}\sim\mathrm{Normal}\left( \eta_{it},\sigma_{e}^{2} \right)$$

Where $y_{it}$ is the under-five mortality rate for country i and year t; $\sigma_{e}^{2}$ is the variance of the measurement error defined by a Gaussian white-noise process (serially and spatially uncorrelated); $b_{0}$ is the intercept; $u_{i}and v_{i}$ are the Besag-York-Mollie (BYM) specification for the spatially structured residual (effect) and unstructured residual (effect), respectively. $\beta$ represents the global time effect and a differential time trend which identifies the interaction between time and space is given as $\delta_{i}$. $\eta_{it}$ is the linear predictor for response Y. For the differential time trend, when the $\delta_{i}$ is less than 0 it signifies the trend is less steep compared to the average trend while $\delta_{i}$ values greater than 0 signifies a steeper trend compared to the average [7,9]

For the non-parametric models, five models with different space-time interaction specifications were built. The first non-parametric model (Model 2) is similar to Bernardinelli et al. (1995) proposed model (Model 1), however, it drops the linearity constraint imposed on the differential temporal trend through a non-parametric formulation for the linear predictor as proposed by Knorr-Held (2000). This can be expressed as:

$\eta_{it}=b_{0}+u_{i}+v_{i}+\gamma_{t}+\phi_{t}$ (2)

Where $b_{0},u_{i,}and v_{i}$ are the same as equation (1) and $\gamma_{t}$ is the temporally structured effect, model using a random walk order of 2 (RW2). RW2 is defined as”

$\gamma_{t}\mid\gamma_{t-1},\gamma_{t-2}\sim\mathrm{Normal}\left( 2\gamma_{t-1}+\gamma_{t-2},\sigma^{2} \right)$ (3)

$\phi_{t}$ is an unstructured temporal effect specified by means of a Gaussian exchangeable prior, given as:

$\phi_{t}\sim\mathrm{Normal}\left( 0,1/\tau_{\phi} \right)$ (4)

The remaining four models are extensions of Model 2 that allow four different types of interactions between space and time as proposed by Knorr-Held (2000). The first space-time interaction (TYPE I) is based on the assumption of unstructured space-time effect; that is, neighbouring spatial units do not affect each other and mortality rates of previous years do not affect that of subsequent years – Model 3. The second space-time interaction (TYPE II) assumes an interaction between a structured temporal effect and an unstructured spatial effect – Model 4. The third space-time interaction (TYPE III) is based on the assumption of an interaction between an unstructured temporal effect and a spatially structured effect – Model 5. Finally, the fourth space-time interaction (TYPE IV) is a complex interaction model that assumes an interaction between spatially and temporally structured effects – Model 6. A detailed discussion of these four space-time interactions and their implementations are described elsewhere [7,9,10].

## Spatiotemporally varying coefficients (STVC) analysis

Next, a Bayesian spatiotemporally varying coefficient (or Bayesian spatial heterogeneity) model to explore spatial and temporal variations in the relationships between the development indicators and under-five mortality rates. Compared to traditional Spatio-temporal models, STVC models move beyond the naïve assumption that the association between predictors and outcome are the same across space and time. It also accounts for autocorrelation in the spatial and temporal dimension of the data; that is, neighbouring units have the potential of affecting the outcome in a unit and mortality rates of previous time periods affect the rate of successive periods. The Bayesian STVC model can be defined simply as:

$$\eta_{i,t}=g(Y_{i,t})=\alpha+f(\boldsymbol{\beta}_{i,t}^{'}\mathbf{x}_{i,t})$$

Where $\eta_{i,t}$ is the linear predictor for response Y (under-five mortality rate) for country i at time t, given *i* = 1, 2 … , n and *t* = 1,2,3, …, T; and g(.) is the appropriate link function. $\alpha$ is the intercept, $\boldsymbol{\beta}_{i,t}^{'}$ is the local coefficient for country i at time t (or the local-scale spatial and temporal coefficients) spatial and temporal, and $\mathbf{x}_{i,t}$ is the covariate matrix for the development indicators in the i-th country at the t-th year. The intrinsic Gaussian Markov random field (IGMRF) for the spatiotemporally varying random effects for estimating the local (country-level) coefficient at each time is given as $f$( ). In this model, we assumed a “besag” model; that is, the spatial random effect is assigned the intrinsic conditional autoregressive (iCAR) distribution for the smoothing of the data according to a defined neighbourhood structure. The iCAR distribution is based on the geographic concept of spatial dependence, that is, the association between the covariates and outcome in a given location (or country) are similar to that of neighbouring locations (or countries) – geographically autocorrelated. An autoregressive model of order 1 was adopted for the temporal structured random effects. A detailed description of the Bayesian STVC model formulation is described elsewhere [11].

Both Spatio-temporal and Spatiotemporally varying coefficients models were implemented using the integrated nested Laplace approximations (INLA) framework with the PARDISO sparse matrix library for high-performance computing [12]. INLA is a computationally less-intensive deterministic algorithm for Bayesian inference based on the latent Gaussian model (LGMs). The default prior specification of R-INLA was used for the distribution of the hyperparameters – a log gamma prior with parameters a = 0.001 and b = 0.001 We assumed a vague prior with parameters a = 1 and b = 0.00005. In the Bayesian models, the parameter estimates were considered significant if the lower and upper 95% credible intervals (Crl) show the same direction for the association. That is, both lower and upper credible intervals are above zero or below zero. All analyses were modelled in the open-access R software [13] using the R-INLA package [14–16]. Results of the Spatio-temporal and spatiotemporally varying coefficient models were also visualised in R software using the tmap and tmaptools packages [17].

## Dealing with missing data

In handling missing information, we conducted sensitivity analyses with two sets of data: original data with missingness and imputed data. In the imputed data we assumed the data were missing completely at random (MCAR), hence employed multiple imputations to address the missing values. The sensitive analyses revealed that there are significant differences in the parameter estimates of the covariates from the two data sets. For instance, in the non-parametric spatio-temporal analyses with the imputed data, some indicators of poor socioeconomic development (namely open defecation) were found to reduce under-five mortality rates. To avoid these biased estimates, we used the original data with some missingness in the outcome and covariates. Table S2 below provides an overview of the missingness in the data. In R-INLA, missing observations in the response variable are treated as unobserved and hence do not contribute to the likelihood. Nonetheless, these unobserved responses can be estimated (computed automatically in R-INLA) by computing the predictive distribution of the corresponding linear predictors and fitted values [18].

| Table S2. Proportion of missingness and number of data points (n=3,510) | | |
| --- | --- | --- |
| **Variables** | % of Missingness | Data Points |
| Mortality rate, under-5 (per 1,000 live births) - U5MR | 1.5 | 3456 |
| Secondary education, pupils (% female) - SEC | 29.2 | 2487 |
| Employment to population ratio, 15+, female (%) (ILO estimate) - FEMP | 8.7 | 3204 |
| Gross domestic product - GDP - per capita (current US$) | 2.3 | 3428 |
| Gross national expenditure (% of GDP) - GNE | 15.7 | 2958 |
| Out-of-pocket expenditure (% of current health expenditure) - OPE | 5.1 | 3332 |
| Current health expenditure (% of GDP) - CHE | 5.1 | 3332 |
| People using at least basic drinking water services (% of population) - WATER | 1.7 | 3449 |
| People practicing open defecation (% of population) - DEF | 2.9 | 3407 |
| People using at least basic sanitation services (% of population) - SANI | 2.0 | 3439 |
| PM2.5 air pollution, mean annual exposure (micrograms per cubic meter) - PM2.5 | 0.0 | 3510 |
| n = total number of observations | | |

## Model Selection

For the Spatio-temporal models, we compared the results of the Bernardinelli et al. (1995) parametric trend for the temporal component and the results for the non-parametric trend models. The optimal model choice for the non-parametric temporal trend models was determined by the deviance information criterion (DIC) and the Watanabe-Akaike information criterion (WAIC) values. DIC and WAIC are criteria for model assessment and model choice. DIC and WAIC as model assessment criteria accounts for both the goodness-of-fit and the complexity of the model through the estimated effective number of parameters [19]. The difference between the two lies in how the effective number of parameters is computed. A detailed description of the DIC and WAIC as measures of model fit can be found elsewhere [9,19,20]. Table 1 below provides the model fit information for the Spatio-temporal models. Similarly, to the Akaike information criterion (AIC), smaller DIC and WAIC values indicate a better model fit for the data. The DIC (17889.79) and WAIC (18108.96) show that for the non-parametric temporal trend models, the model with Type IV (Model 6) space-time interaction is a better fit or statistically preferable compared to the others. The DIC and WAIC values also show that the parametric temporal trend model (Model 1) is a better fit than all non-parametric temporal trend models, except Model 6 (model with Type IV space-time interaction).

| Table S3. Diagnostics information for space-time interaction model selection | | |
| --- | --- | --- |
|  | DIC | WAIC |
| Model 1 - Parametric model (Bernardinelli model) | 18657.42 | 18897.03 |
| Model 2 - Non-parametric temporal effect | 25563.05 | 25588.74 |
| Model 3 - TYPE I | 25211.67 | 25353.74 |
| Model 4 - TYPE II | 38085.58 | 38085.57 |
| Model 5 - TYPE III | 25604.86 | 25680.23 |
| Model 6 - TYPE IV | 17889.79 | 18108.96 |
|  | | |

# Appendix C – Additional Figures


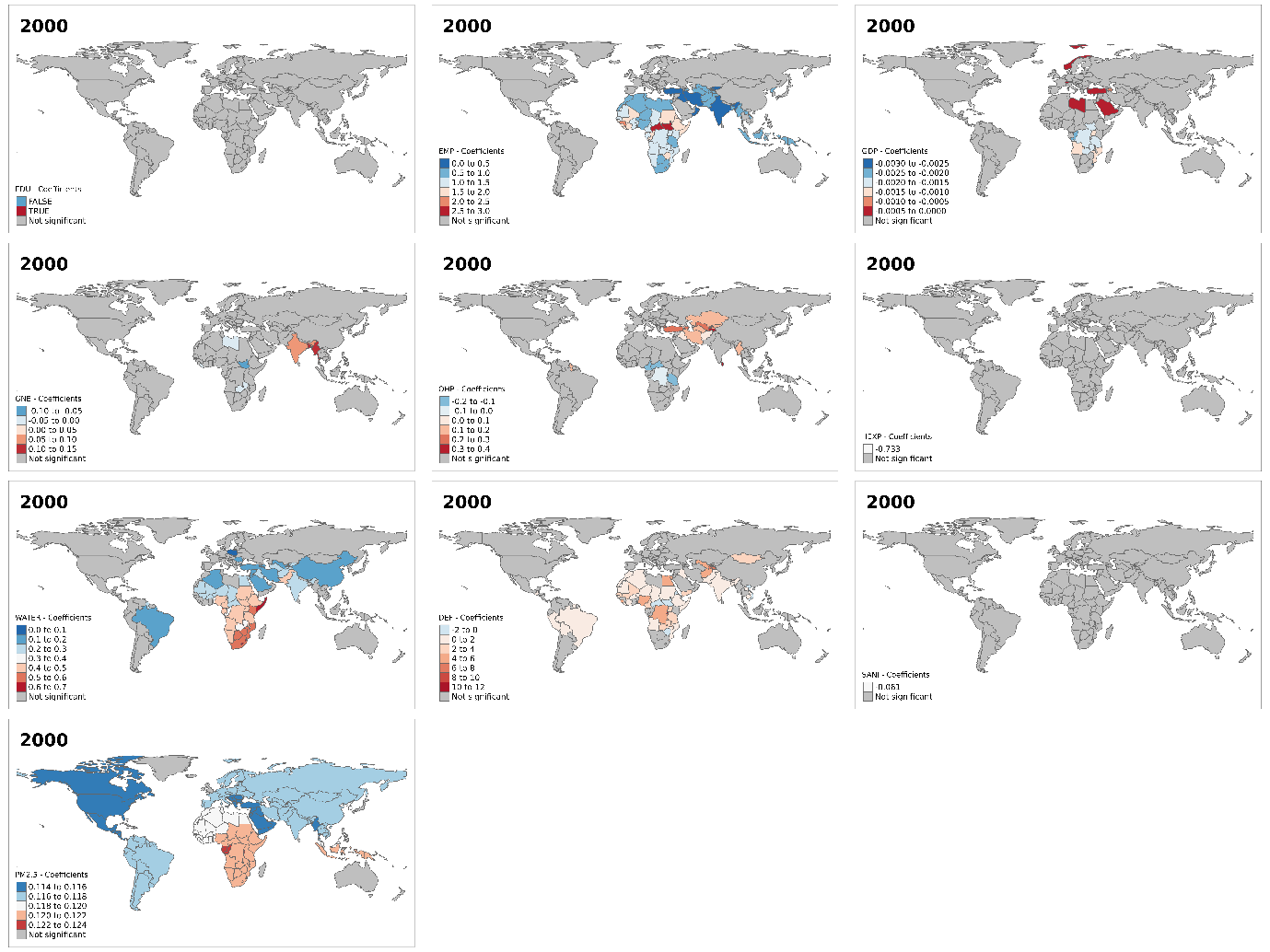


Figure S1. Spatial varying coefficients for the effects of development indicators on under-five mortality rates in the year 2000 showing countries with significant observed effects (95% Credible Interval). EDU (Secondary education, pupils (% female)); EMP (Employment to population ratio, 15+, female (%) (ILO estimate)); GDP (Gross domestic product - per capita (current US$)); GNE (Gross national expenditure (% of GDP)); OHP (Out-of-pocket expenditure (% of current health expenditure)); HEXP (Current health expenditure (% of GDP)); WATER (People using at least basic drinking water services (% of population)); DEF (People practicing open defecation (% of population)); SANI (People using at least basic sanitation services (% of population)); PM (PM2.5 air pollution, mean annual exposure (micrograms per cubic meter))


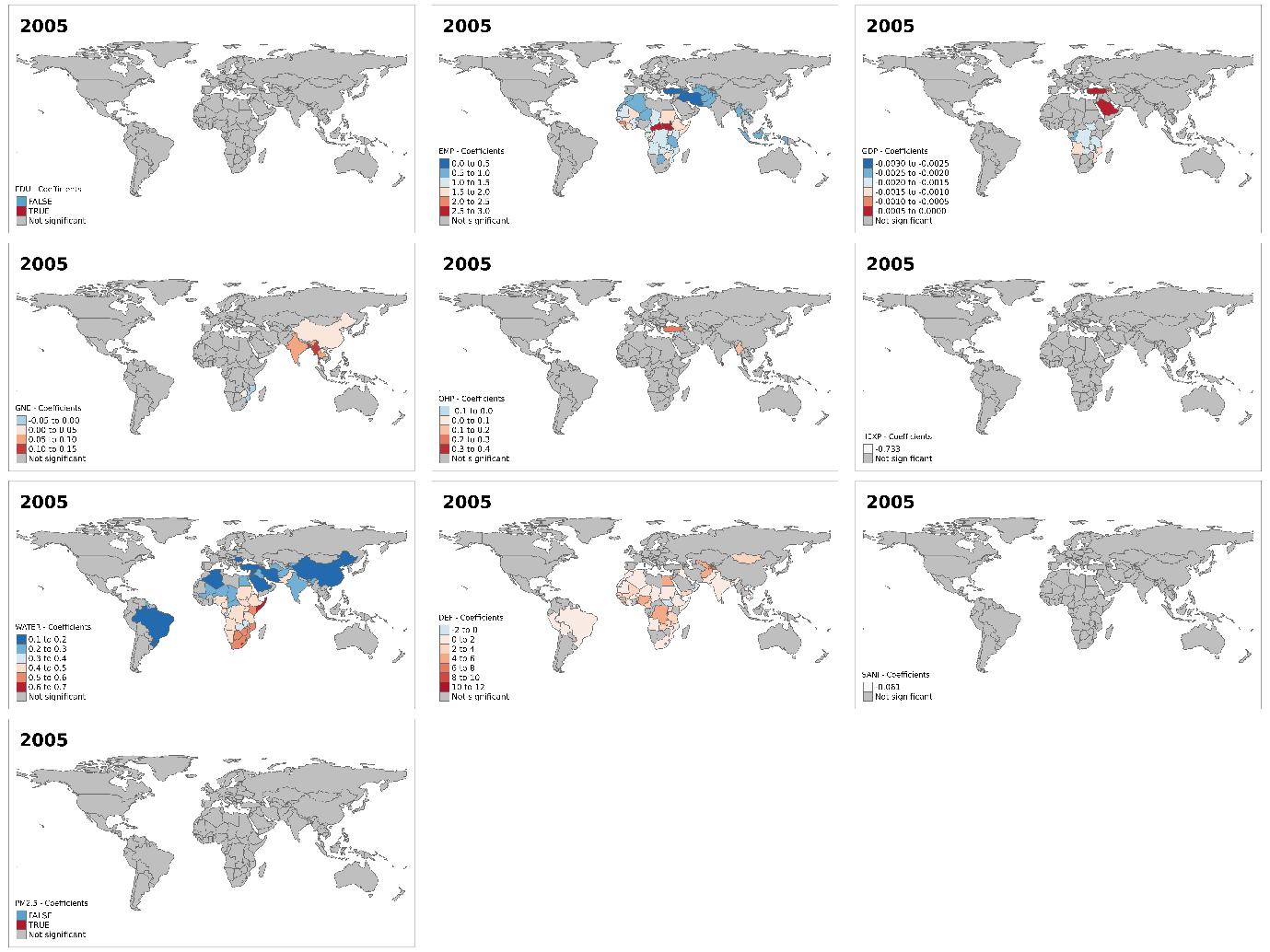


Figure S2. Spatial varying coefficients for the effects of development indicators on under-five mortality rates in the year 2005 showing countries with significant observed effects (95% Credible Interval). EDU (Secondary education, pupils (% female)); EMP (Employment to population ratio, 15+, female (%) (ILO estimate)); GDP (Gross domestic product - per capita (current US$)); GNE (Gross national expenditure (% of GDP)); OHP (Out-of-pocket expenditure (% of current health expenditure)); HEXP (Current health expenditure (% of GDP)); WATER (People using at least basic drinking water services (% of population)); DEF (People practicing open defecation (% of population)); SANI (People using at least basic sanitation services (% of population)); PM (PM2.5 air pollution, mean annual exposure (micrograms per cubic meter))


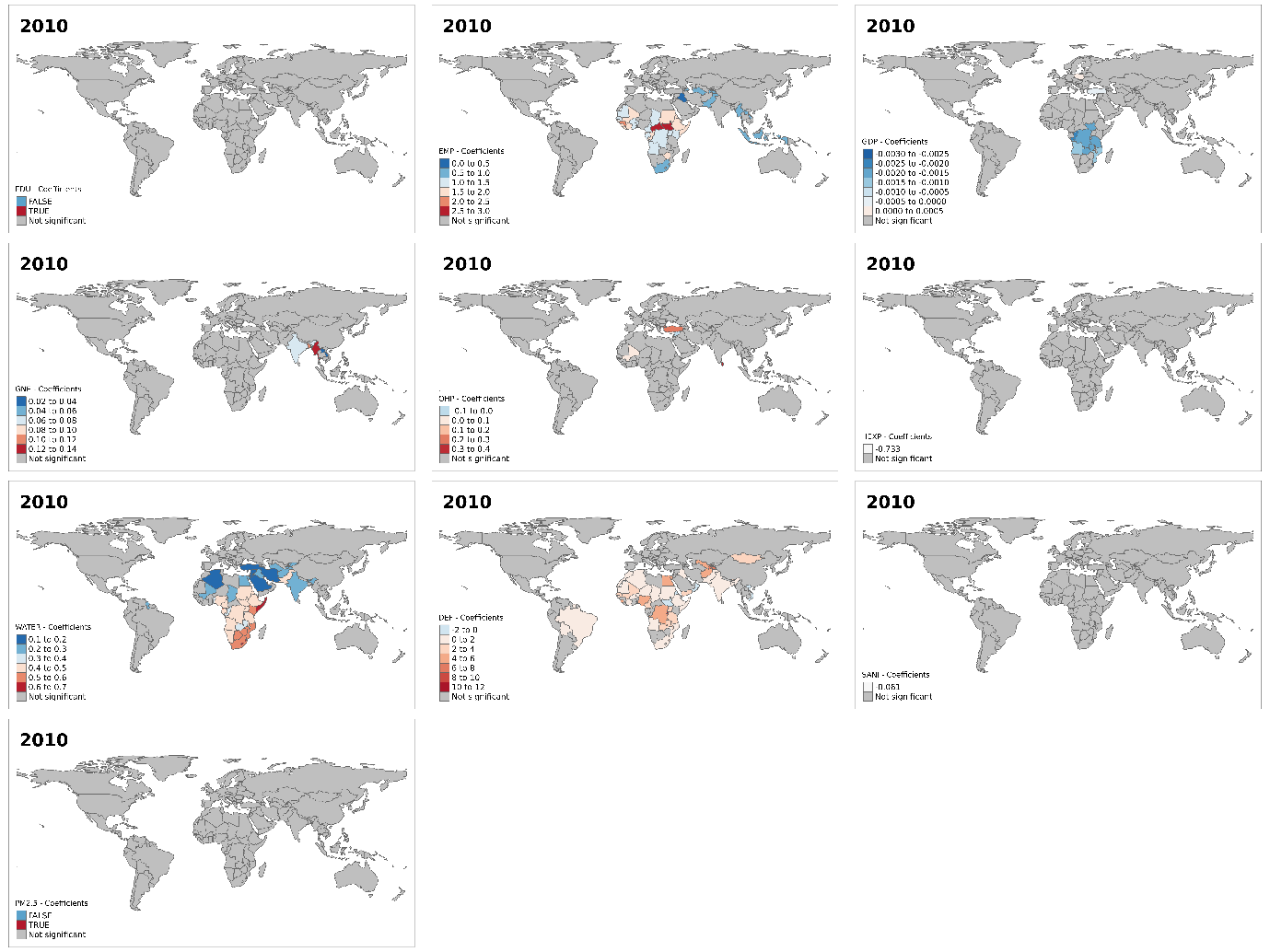


Figure S3. Spatial varying coefficients for the effects of development indicators on under-five mortality rates in the year 2010 showing countries with significant observed effects (95% Credible Interval). EDU (Secondary education, pupils (% female)); EMP (Employment to population ratio, 15+, female (%) (ILO estimate)); GDP (Gross domestic product - per capita (current US$)); GNE (Gross national expenditure (% of GDP)); OHP (Out-of-pocket expenditure (% of current health expenditure)); HEXP (Current health expenditure (% of GDP)); WATER (People using at least basic drinking water services (% of population)); DEF (People practicing open defecation (% of population)); SANI (People using at least basic sanitation services (% of population)); PM (PM2.5 air pollution, mean annual exposure (micrograms per cubic meter))


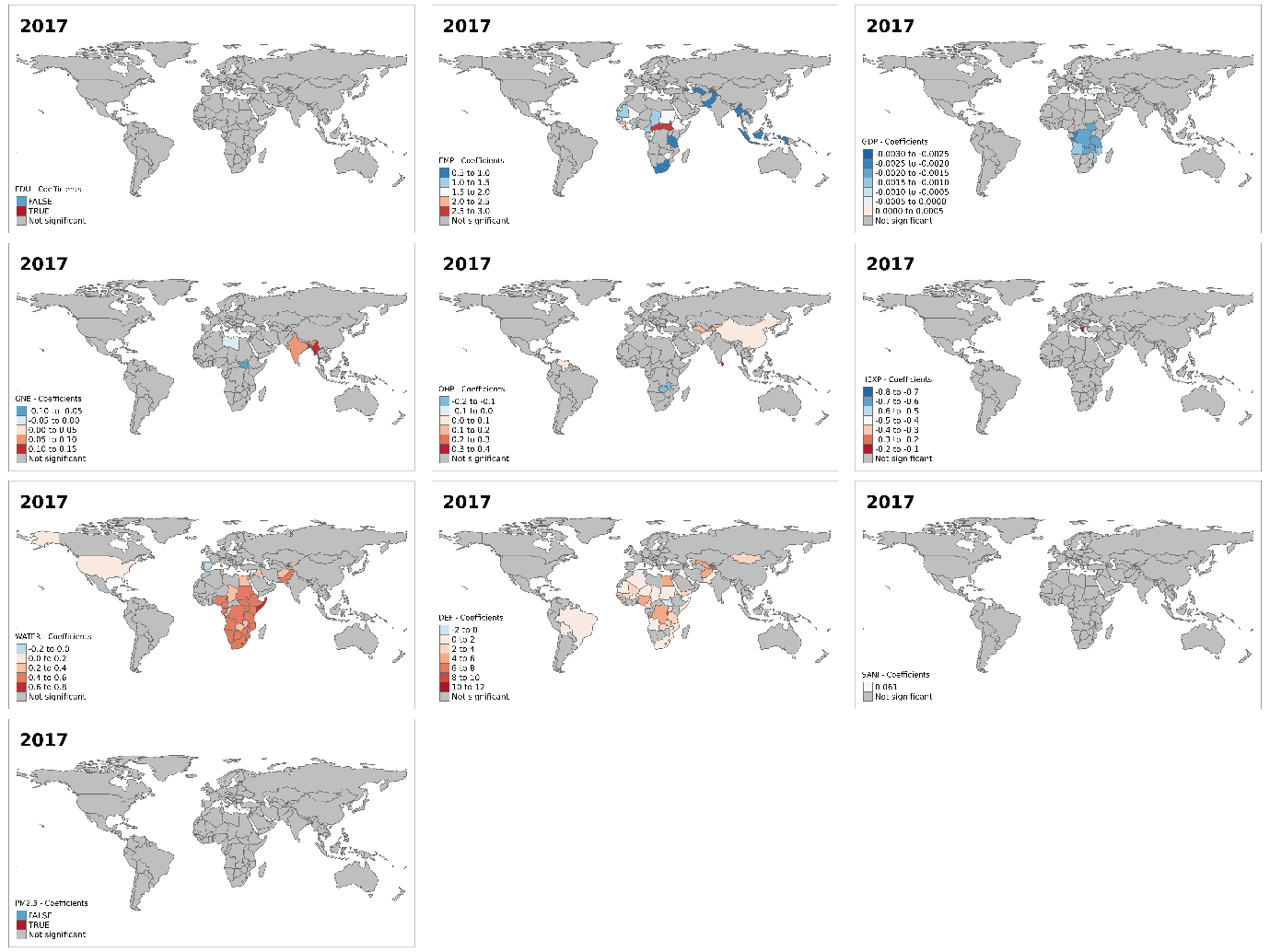


Figure S4. Spatial varying coefficients for the effects of development indicators on under-five mortality rates in the year 2017 showing countries with significant observed effects (95% Credible Interval). EDU (Secondary education, pupils (% female)); EMP (Employment to population ratio, 15+, female (%) (ILO estimate)); GDP (Gross domestic product - per capita (current US$)); GNE (Gross national expenditure (% of GDP)); OHP (Out-of-pocket expenditure (% of current health expenditure)); HEXP (Current health expenditure (% of GDP)); WATER (People using at least basic drinking water services (% of population)); DEF (People practicing open defecation (% of population)); SANI (People using at least basic sanitation services (% of population)); PM (PM2.5 air pollution, mean annual exposure (micrograms per cubic meter))

# Appendix D – Missing Information from 1960 to 2018 by Study Variables

| Table S4. Number of countries with missing information from 1960 to 2019 by study variables Number of countries with missing data for all available years by the study variables | | | | | | | | | | | |
| --- | --- | --- | --- | --- | --- | --- | --- | --- | --- | --- | --- |
| Year | U5MR | EDU | EMP | GDP | GNE | OHP | HEXP | WATER | DEF | SANI | PM |
| 1960 | 98 | 195 | 195 | 99 | 135 | 195 | 195 | 195 | 195 | 195 | 195 |
| 1961 | 96 | 195 | 195 | 98 | 132 | 195 | 195 | 195 | 195 | 195 | 195 |
| 1962 | 94 | 195 | 195 | 95 | 131 | 195 | 195 | 195 | 195 | 195 | 195 |
| 1963 | 86 | 195 | 195 | 95 | 131 | 195 | 195 | 195 | 195 | 195 | 195 |
| 1964 | 80 | 195 | 195 | 95 | 130 | 195 | 195 | 195 | 195 | 195 | 195 |
| 1965 | 78 | 195 | 195 | 88 | 126 | 195 | 195 | 195 | 195 | 195 | 195 |
| 1966 | 76 | 195 | 195 | 86 | 124 | 195 | 195 | 195 | 195 | 195 | 195 |
| 1967 | 72 | 195 | 195 | 83 | 122 | 195 | 195 | 195 | 195 | 195 | 195 |
| 1968 | 68 | 195 | 195 | 81 | 121 | 195 | 195 | 195 | 195 | 195 | 195 |
| 1969 | 61 | 195 | 195 | 81 | 120 | 195 | 195 | 195 | 195 | 195 | 195 |
| 1970 | 58 | 157 | 195 | 72 | 99 | 195 | 195 | 195 | 195 | 195 | 195 |
| 1971 | 53 | 76 | 195 | 71 | 100 | 195 | 195 | 195 | 195 | 195 | 195 |
| 1972 | 50 | 82 | 195 | 71 | 99 | 195 | 195 | 195 | 195 | 195 | 195 |
| 1973 | 50 | 88 | 195 | 71 | 99 | 195 | 195 | 195 | 195 | 195 | 195 |
| 1974 | 46 | 89 | 195 | 70 | 99 | 195 | 195 | 195 | 195 | 195 | 195 |
| 1975 | 42 | 97 | 195 | 68 | 95 | 195 | 195 | 195 | 195 | 195 | 195 |
| 1976 | 40 | 89 | 195 | 67 | 92 | 195 | 195 | 195 | 195 | 195 | 195 |
| 1977 | 38 | 88 | 195 | 64 | 91 | 195 | 195 | 195 | 195 | 195 | 195 |
| 1978 | 34 | 94 | 195 | 65 | 91 | 195 | 195 | 195 | 195 | 195 | 195 |
| 1979 | 31 | 96 | 195 | 64 | 93 | 195 | 195 | 195 | 195 | 195 | 195 |
| 1980 | 28 | 96 | 195 | 54 | 87 | 195 | 195 | 195 | 195 | 195 | 195 |
| 1981 | 22 | 92 | 195 | 51 | 84 | 195 | 195 | 195 | 195 | 195 | 195 |
| 1982 | 19 | 97 | 195 | 51 | 83 | 195 | 195 | 195 | 195 | 195 | 195 |
| 1983 | 17 | 94 | 195 | 50 | 80 | 195 | 195 | 195 | 195 | 195 | 195 |
| 1984 | 13 | 93 | 195 | 49 | 80 | 195 | 195 | 195 | 195 | 195 | 195 |
| 1985 | 3 | 96 | 195 | 47 | 79 | 195 | 195 | 195 | 195 | 195 | 195 |
| 1986 | 3 | 85 | 195 | 46 | 78 | 195 | 195 | 195 | 195 | 195 | 195 |
| 1987 | 3 | 92 | 195 | 42 | 76 | 195 | 195 | 195 | 195 | 195 | 195 |
| 1988 | 3 | 91 | 195 | 39 | 75 | 195 | 195 | 195 | 195 | 195 | 195 |
| 1989 | 3 | 94 | 195 | 39 | 73 | 195 | 195 | 195 | 195 | 195 | 195 |
| 1990 | 3 | 95 | 195 | 23 | 62 | 195 | 195 | 195 | 195 | 195 | 8 |
| 1991 | 3 | 92 | 17 | 24 | 60 | 195 | 195 | 195 | 195 | 195 | 195 |
| 1992 | 3 | 93 | 17 | 24 | 59 | 195 | 195 | 195 | 195 | 195 | 195 |
| 1993 | 3 | 86 | 17 | 21 | 55 | 195 | 195 | 195 | 195 | 195 | 195 |
| 1994 | 3 | 91 | 17 | 20 | 53 | 195 | 195 | 195 | 195 | 195 | 195 |
| 1995 | 3 | 99 | 17 | 14 | 47 | 195 | 195 | 195 | 195 | 195 | 8 |
| 1996 | 3 | 104 | 17 | 14 | 47 | 195 | 195 | 195 | 195 | 195 | 195 |
| 1997 | 3 | 119 | 17 | 14 | 45 | 195 | 195 | 195 | 195 | 195 | 195 |
| 1998 | 3 | 107 | 17 | 14 | 45 | 195 | 195 | 195 | 195 | 195 | 195 |
| 1999 | 3 | 56 | 17 | 13 | 45 | 195 | 195 | 195 | 195 | 195 | 195 |
| 2000 | 3 | 59 | 17 | 7 | 38 | 13 | 13 | 10 | 15 | 11 | 8 |
| 2001 | 3 | 61 | 17 | 6 | 37 | 13 | 13 | 7 | 10 | 8 | 195 |
| 2002 | 3 | 62 | 17 | 5 | 35 | 12 | 12 | 5 | 8 | 6 | 195 |
| 2003 | 3 | 65 | 17 | 5 | 34 | 10 | 10 | 5 | 8 | 6 | 195 |
| 2004 | 3 | 53 | 17 | 4 | 33 | 9 | 9 | 5 | 8 | 6 | 195 |
| 2005 | 3 | 51 | 17 | 4 | 32 | 9 | 9 | 3 | 7 | 5 | 8 |
| 2006 | 3 | 59 | 17 | 4 | 30 | 9 | 9 | 2 | 3 | 3 | 195 |
| 2007 | 3 | 51 | 17 | 4 | 29 | 9 | 9 | 2 | 2 | 2 | 195 |
| 2008 | 3 | 51 | 17 | 4 | 27 | 9 | 9 | 2 | 3 | 2 | 195 |
| 2009 | 3 | 54 | 17 | 4 | 28 | 9 | 9 | 2 | 3 | 2 | 195 |
| 2010 | 3 | 53 | 17 | 3 | 27 | 8 | 8 | 2 | 3 | 2 | 8 |
| 2011 | 3 | 49 | 17 | 3 | 27 | 8 | 8 | 1 | 2 | 1 | 8 |
| 2012 | 3 | 57 | 17 | 4 | 28 | 9 | 9 | 1 | 2 | 1 | 8 |
| 2013 | 3 | 66 | 17 | 4 | 29 | 10 | 10 | 1 | 2 | 1 | 8 |
| 2014 | 3 | 58 | 17 | 4 | 28 | 10 | 10 | 2 | 3 | 2 | 8 |
| 2015 | 3 | 54 | 17 | 5 | 29 | 10 | 10 | 2 | 5 | 2 | 8 |
| 2016 | 3 | 58 | 17 | 6 | 30 | 11 | 11 | 3 | 7 | 4 | 8 |
| 2017 | 3 | 62 | 17 | 6 | 31 | 10 | 10 | 6 | 12 | 7 | 8 |
| 2018 | 3 | 123 | 17 | 8 | 37 | 195 | 195 | 195 | 195 | 195 | 195 |
| Abbreviations: U5MR = Under-five mortality rate per 1,000 live births EDU = Secondary education, pupils (% female)  EMP = Employment to population ratio, 15+, female (%) (ILO estimate)  GDP = Gross domestic product - per capita (current US$) GNE = Gross national expenditure (% of GDP)  OHP = Out-of-pocket expenditure (% of current health expenditure)  HEXP = Current health expenditure (% of GDP)  WATER = People using at least basic drinking water services (% of population)  DEF = People practicing open defecation (% of population)  SANI = People using at least basic sanitation services (% of population)  PM = PM2.5 air pollution, mean annual exposure (micrograms per cubic meter) | | | | | | | | | | | |

# Reference

1. Alkema L, New JR. Global estimation of child mortality using a Bayesian B-spline bias-reduction model. Ann Appl Stat. 2014;8:2122–49.

2. Pollack CE, Chideya S, Cubbin C, Williams B, Dekker M, Braveman P. Should Health Studies Measure Wealth?. A Systematic Review. Am J Prev Med. 2007;33:250–64.

3. Cole WM. Wealth and health revisited: Economic growth and wellbeing in developing countries, 1970 to 2015. Soc Sci Res [Internet]. Elsevier; 2019;77:45–67. Available from: https://doi.org/10.1016/j.ssresearch.2018.09.003

4. Cameron L, Williams J. Is the relationship between socioeconomic status and health stronger for older children in developing countries? Demography. 2009;46:303–24.

5. Bloom DE, Canning D. The Health and Wealth of Nations. Science (80- ) [Internet]. 2000;287:1207–9. Available from: https://www.science.org/doi/abs/10.1126/science.287.5456.1207

6. Aber JL, Bennett NG, Conley DC, Li J. The effects of poverty on child health and development. Annu Rev Public Health. 1997;18:463–83.

7. Blangiardo M, Cameletti M, Baio G, Rue H. Spatial and spatio-temporal models with R-INLA. Spat Spatiotemporal Epidemiol. Elsevier Ltd; 2013;7:39–55.

8. Bernardinelli L, Clayton D, Pascutto C, Montomoli C, Ghislandi M, Songini M. Bayesian analysis of space—time variation in disease risk. Stat Med. 1995;14:2433–43.

9. Blangiardo M, Cameletti M. Spatial and Spatio-temporal Bayesian Models with R-INLA. John Wiley & Sons; 2015.

10. Knorr-Held L. Bayesian modelling of inseparable space-time variation in disease risk. Stat Med. 2000;19:2555–67.

11. Song C, Wang Y, Yang X, Yang Y, Tang Z, Wang X, et al. Spatial and temporal impacts of socioeconomic and environmental factors on healthcare resources: A county-level bayesian local spatiotemporal regression modeling study of hospital beds in Southwest China. Int J Environ Res Public Health. 2020;17:1–23.

12. van Niekerk J, Bakka H, Rue H, Schenk O. New Frontiers in Bayesian Modeling Using the INLA Package in R . J Stat Softw. 2021;100.

13. R Core Team. R: A language and environment for statistical computing. Vienna, Austria: R Foundation for Statistical Computing; 2020.

14. Bakka H, Fuglstad G, Riebler A, Bolin D, Krainski E, Simpson D, et al. Spatial modelling with R-INLA : A review. 2018.

15. Rue H, Martino S, Chopin N. Approximate Bayesian inference for latent Gaussian models by using integrated nested Laplace approximations. J R Stat Soc Ser B Stat Methodol. 2009;71:319–92.

16. Martino S, Akerkar R, Rue H. Approximate Bayesian Inference for Survival Models. Scand J Stat. 2011;38:514–28.

17. Tennekes M. tmap : Thematic Maps in R. J Stat Softw. 2018;84:1–39.

18. Krainski ET, Gómez-Rubio V, Bakka H, Lenzi A, Castro-Camilo D, Simpson D, et al. Advanced Spatial Differential Equations Stochastic Partial Modeling with Using R and INLA. New York: Chapman and Hall/CRC; 2019.

19. Spiegelhalter DJ, Best NG, Carlin BP, Van Der Linde A. Bayesian measures of model complexity and fit. J R Stat Soc Ser B Stat Methodol. 2002;64:583–616.

20. Watanabe S. Asymptotic equivalence of Bayes cross validation and widely applicable information criterion in singular learning theory. J Mach Learn Res. 2010;11:3571–94.
